# Targeting PI3K**γ** anchoring enhances CFTR membrane localization and modulator efficacy via PKD1

**DOI:** 10.1172/jci.insight.198846

**Published:** 2026-03-23

**Authors:** Alessandra Murabito, Marco Mergiotti, Valeria Capurro, Alessia Loffreda, Mingchuan Li, Paola Peretto, Kai Ren, Andrea Raimondi, Carlo Tacchetti, Dario Diviani, Nicoletta Pedemonte, Emilio Hirsch, Alessandra Ghigo

**Affiliations:** 1 Department of Molecular Biotechnology and Health Sciences, Molecular Biotechnology Center “Guido Tarone”, University of Torino, Torino, Italy.; 2 UOC Genetica Medica, IRCCS Istituto Giannina Gaslini, Genova, Italy.; 3 Experimental Imaging Center, IRCCS Ospedale San Raffaele, Milan, Italy.; 4 Università Vita-Salute San Raffaele, Milan, Italy.; 5 Department of Biomedical Sciences, Faculty of Biology and Medicine, University of Lausanne; Lausanne, Switzerland.; 6 Kither Biotech, Torino, Italy.

**Keywords:** Cell biology, Pulmonology, Genetic diseases, Peptides, Therapeutics

## Abstract

Mutations in the cystic fibrosis transmembrane conductance regulator (*CFTR*) gene, which encodes a cAMP-activated chloride channel, cause cystic fibrosis (CF), the most common life-threatening inherited disorder among White individuals. Current CFTR correctors and potentiators, such as elexacaftor-tezacaftor-ivacaftor (ETI), only partially restore the function of the most prevalent mutant, F508del-CFTR, resulting in residual disease in people with CF. Here, we demonstrate that a mimetic peptide targeting the A-kinase–anchoring protein (AKAP) function of PI3Kγ (PI3Kγ MP), and driving localized cAMP elevation, enhances F508del-CFTR membrane localization, maximizing ETI efficacy in restoring chloride secretion. Mechanistically, PI3Kγ MP activates an AKAP-Lbc–anchored pool of PKD1, a known regulator of membrane trafficking. Consistently, PKD1 inhibition prevents PI3Kγ MP from enhancing the membrane expression of ETI-corrected F508del-CFTR. Overall, our findings reveal a regulatory pathway controlling CFTR membrane abundance via the AKAP function of PI3Kγ, which can be targeted to overcome the limitations of current CFTR modulator therapies.

## Abstract 2

A peptide targeting PI3Kγ boosts airway CFTR membrane localization, offering a new strategy to enhance the efficacy cystic fibrosis therapies

## Graphical abstract

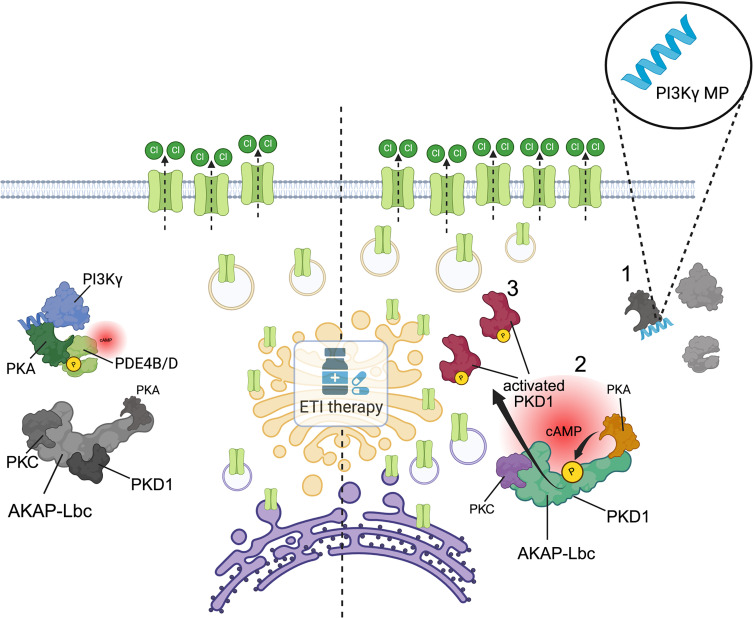

## Introduction

Cystic fibrosis (CF) is the most common life-threatening inherited disorder among the White population. It is caused by mutations in the cystic fibrosis transmembrane conductance regulator (*CFTR*) gene, which encodes a cAMP-activated anion channel expressed on the surface of epithelial cells, regulating mucus hydration and clearance (1). CFTR dysfunction results in impaired chloride (Cl^−^) and water secretion across the airway epithelium, producing a viscous mucus that traps pathogens, causing chronic infections and inflammation (2). These complications are responsible for a progressive decline in airway function, ultimately leading to respiratory failure (3).

In the United States, about 85% of people with CF carry at least 1 copy of the F508del allele, resulting in a mutant CFTR channel, F508del-CFTR, characterized by defective folding, impaired gating, and reduced stability at the plasma membrane (1). The standard of care for patients carrying at least 1 F508del allele is a combination of 2 “correctors,” elexacaftor (VX-445) and tezacaftor (VX-661), and the “potentiator” ivacaftor (VX-770), which correct the folding and improve the gating of the mutant channel, respectively (1). Despite its high clinical efficacy, resulting in an average lung function improvement of 9.8% in F508del heterozygous patients after 6 months of treatment (4), the triple-combination therapy of elexacaftor-tezacaftor-ivacaftor (ETI) restores F508del-CFTR activity to only 50% of that of the WT channel (5). As a result, patients with CF on ETI therapy continue to experience residual mucus dysfunction, airway infection, and inflammation (6–8), underscoring the need for strategies to enhance treatment efficacy.

Boosting cAMP signaling enhances the efficacy of CFTR modulators by activating PKA, which phosphorylates CFTR, a step essential for optimal potentiation by VX-770 (9, 10). On the other hand, cAMP can enhance the effectiveness of correctors like lumacaftor (VX-809) in rescuing F508del-CFTR, by tethering the channel to the actin cytoskeleton and reducing its endocytosis (11, 12). Hence, agents that elevate cAMP near the F508del-CFTR may help to prolong the residence time of the channel at the plasma membrane, ultimately enhancing the efficacy of CFTR modulator therapies (13).

Our previous research demonstrated that a physiological and compartment-restricted increase in cAMP near the CFTR can be achieved by targeting the A-kinase–anchoring protein (AKAP) function of PI3Kγ. We showed that a PI3Kγ mimetic peptide (PI3Kγ MP) disrupts the interaction between PI3Kγ and PKA. This disruption inhibits a specific phosphodiesterase 4 (PDE4) pool responsible for hydrolyzing cAMP in the vicinity of CFTR, thereby enhancing PKA-mediated phosphorylation and gating of the channel. Accordingly, PI3Kγ MP promotes the opening of WT CFTR and augments the efficacy of the VX-770 potentiator in rescuing the function of F508del-CFTR (10). However, the role of the PI3Kγ/PKA signalosome in regulating CFTR membrane expression has remained unexplored.

Here, we demonstrate that PI3Kγ MP increases the plasma membrane density of F508del-CFTR, thereby enhancing the efficacy of the CFTR modulator combination ETI. This effect is mediated by PKD1, which acts downstream of cAMP elevation and PKA activation induced by PI3Kγ MP. Overall, these findings reveal a regulatory mechanism governing CFTR membrane expression that can be harnessed to enhance the efficacy of current CFTR modulator therapies.

## Results

### PI3Kγ MP increases the plasma membrane density of F508del-CFTR in combination with ETI therapy.

**Figure 1:**
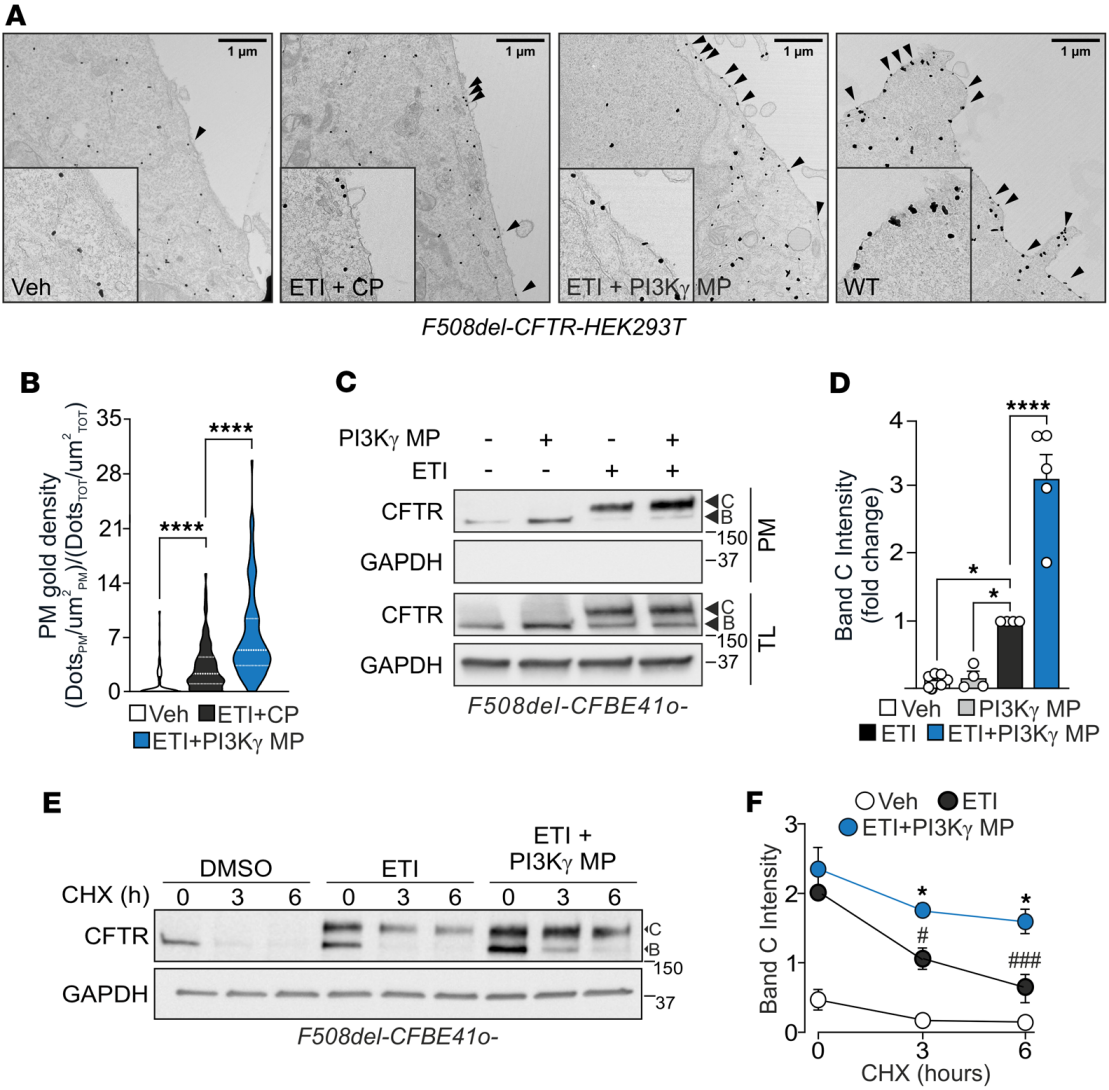
PI3Kγ MP increases the plasma membrane density of F508del-CFTR in combination with ETI. (**A**) Representative immunogold electron microscopy images showing the distribution of overexpressed F508del-CFTR-GFP in HEK293T cells. Cells were treated for 24 hours with DMSO (Veh), ETI (3 μM VX-445, 10 μM VX-661, and 1 μM VX-770) plus control peptide (ETI + 25 μM CP), or ETI plus PI3Kγ MP (ETI + 25 μM PI3Kγ MP). HEK293T cells expressing WT CFTR-GFP served as positive controls. Arrowheads: CFTR channels located at the plasma membrane. Insets: higher magnification of selected regions. Scale bar: 1 μm. (**B**) Quantification of plasma membrane gold density as shown in **A**; 99–118 cells from 19–20 images were quantified. ****P < 0.0001 by Kruskal-Wallis test with Dunn’s multiple-comparison test. (**C**) Representative Western blot of CFTR and GAPDH in plasma membrane fractions and total lysates (TLs) of F508del-CFBE41o- cells treated for 24 hours with ETI alone or ETI + PI3Kγ MP (25 μM). GAPDH was absent in plasma membrane fractions and used as a loading control in TLs. (**D**) Quantification of plasma membrane CFTR band C intensity as shown in **C**, expressed as fold-change relative to ETI alone. *n* = 4–6. *P < 0.05, ****P < 0.0001 by 1-way ANOVA with Dunnett’s multiple-comparison test. (**E**) Representative Western blot of CFTR and GAPDH levels in F508del-CFBE41o- cells treated for 24 hours with ETI alone or ETI + PI3Kγ MP (25 μM) and cycloheximide (CHX, 1 μg/mL) exposure. Cells were lysed at the indicated times after CHX treatment. (**F**) Quantification of CFTR band C as shown in **E**, expressed as fold-change relative to t = 0. *n* = 3. ^##^*P* < 0.01, ^###^*P* < 0.001 versus t = 0 by 1-way ANOVA with Bonferroni’s post hoc test; *P < 0.05 for ETI versus ETI + PI3Kγ MP by Welch’s *t* test. Data shown as mean ± SEM. *n*: number of independent experiments. Data points represent independent biological replicates.

First, we assessed the extent to which PI3Kγ MP influences CFTR membrane localization and augments the effect of ETI on the surface expression of F508del-CFTR. Immunogold labeling in HEK293T cells expressing GFP-tagged F508del-CFTR revealed that ETI treatment in combination with PI3Kγ MP resulted in a 2-fold increase in F508del-CFTR at the plasma membrane compared with ETI administered with the inactive control peptide (CP) (Figure 1, A and B). These findings were corroborated in F508del-CFBE41o- cells using surface protein biotinylation followed by streptavidin pulldown, which showed a 3-fold increase in mature (C band) F508del-CFTR at the cell surface in cells treated with PI3Kγ MP plus ETI compared with ETI alone (Figure 1, C and D). Notably, global cAMP elevation induced by the cell-permeable, non-hydrolysable analog CPTcAMP failed to phenocopy the effect of the peptide (Supplemental Figure 1, A and B; supplemental material available online with this article; https://doi.org/10.1172/jci.insight.198846DS1), suggesting that the ability of PI3Kγ MP to increase the plasma membrane abundance of F508del-CFTR depends on its capacity to elevate cAMP locally (10). Enhanced trafficking of CFTR to the plasma membrane may increase its functional lifetime by improving its membrane stability and reducing degradation (14, 15). Consistent with this, cycloheximide (CHX) chase experiments showed that, 6 hours after inhibition of de novo protein synthesis, the level of mature F508del-CFTR in CFBE41o- cells treated with both ETI and PI3Kγ MP was 47% higher than in cells treated with ETI alone (Figure 1, E and F). Altogether, these data indicate that PI3Kγ MP enhances the plasma membrane density of F508del-CFTR under ETI therapy.

### PI3Kγ MP does not promote F508del-CFTR maturation.

**Figure 2:**
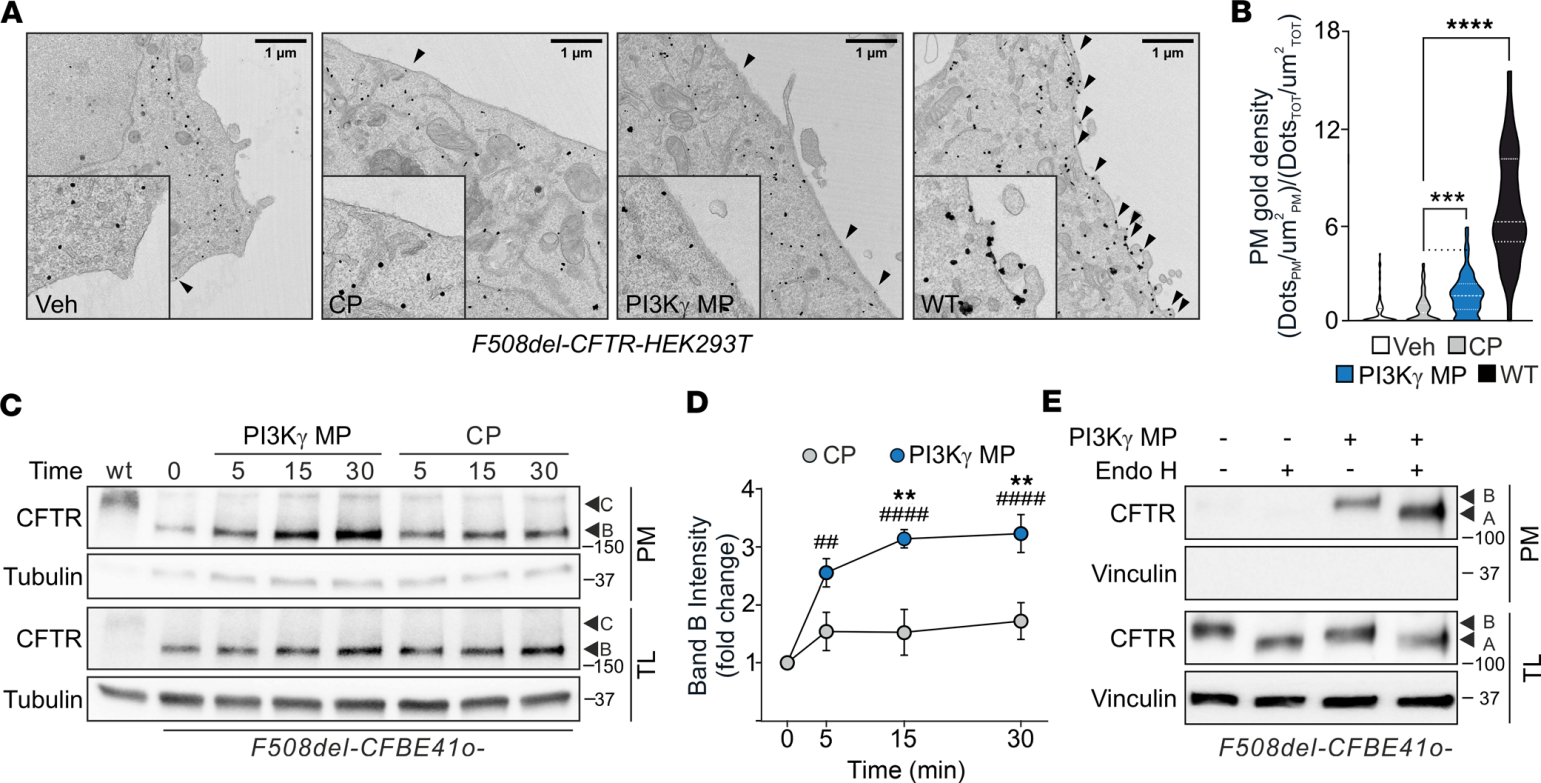
PI3Kγ MP does not affect F508del-CFTR maturation. (**A**) Representative immunogold electron microscopy images showing the distribution of overexpressed F508del-CFTR-GFP in HEK293T cells after 15 minutes of treatment with DMSO (Veh), control peptide (CP; 25 μM), or PI3Kγ MP (25 μM). HEK293T cells expressing WT-CFTR-GFP served as positive controls. Arrowheads indicate CFTR channels located at the plasma membrane. Insets: Higher magnification of selected regions. Scale bar: 1 μm. (**B**) Quantification of plasma membrane gold density as shown in **A**. For Veh, CP, and PI3Kγ MP, 68 cells were quantified; 32 cells were quantified for WT. ***P < 0.001, ****P < 0.0001 by Kruskal-Wallis test with Dunn’s multiple-comparison test. (**C**) Representative Western blot of CFTR and tubulin in plasma membrane fractions and total lysates (TLs) of F508del-CFBE41o- cells treated with CP or PI3Kγ MP for 5, 15, or 30 minutes. WT-CFTR-CFBE41o- cells served as controls for mature complex-glycosylated CFTR (band C). Tubulin was absent in plasma membrane fractions and used as a loading control in TLs. (**D**) Quantification of immature core-glycosylated CFTR (band B) in plasma membrane fractions as in **C**. *n* = 5. ^##^*P* < 0.01, ^####^*P* < 0.0001 versus t = 0, and **P < 0.01 versus CP by 1-way ANOVA with Bonferroni’s post hoc test. (**E**) Representative Western blot of CFTR and vinculin in plasma membrane fractions and TLs from F508del-CFBE41o- cells treated with PI3Kγ MP (25 μM; 15 minutes). Plasma membrane proteins were biotinylated, isolated with streptavidin beads, and incubated with Endo H for 1 hour. Vinculin was absent in plasma membrane fractions and used as a loading control in TLs. Throughout, data are mean ± SEM. *n*: number of independent experiments. Data points represent independent biological replicates.

Next, we investigated the mechanism by which PI3Kγ MP enhances the plasma membrane density of F508del-CFTR. To assess whether the peptide promotes maturation of the mutant channel, we performed immunogold staining in GFP-F508del-CFTR–expressing HEK293T cells in the absence of correctors, thereby excluding any confounding effects from pharmacological chaperones. This analysis revealed a 2-fold increase in CFTR membrane density in cells treated with PI3Kγ MP compared with those treated with CP, reaching approximately 20% of the levels observed in cells expressing WT CFTR (Figure 2, A and B). Cell-surface biotinylation experiments in F508del-CFBE41o- cells confirmed a significant increase in CFTR levels at the cell membrane at 15 and 30 minutes after treatment with PI3Kγ MP compared with CP (Figure 2, C and D). Notably, PI3Kγ MP increased the membrane abundance of the immature core-glycosylated (B band) but not the mature complex-glycosylated (C band) CFTR (Figure 2, C and D). These observations suggest that the peptide does not replicate the mechanism of CFTR correctors in rescuing the folding and defective trafficking of F508del-CFTR from the endoplasmic reticulum (ER) to the Golgi and plasma membrane. As further confirmation, we treated biotin-labeled cell lysates with endoglycosidase H (Endo H), an enzyme that removes ER core-glycosylation but not complex sugars added in the Golgi. After Endo H treatment, a shift in the molecular weight of F508del-CFTR was observed in CFBE41o- cells treated with the peptide (Figure 2E), indicating that the CFTR at the membrane was sensitive to Endo H and therefore in its immature, ER core-glycosylated form. Further supporting the inability of PI3Kγ MP to promote F508del-CFTR maturation, neither the cAMP analog CPTcAMP nor the CFTR potentiator VX-770 elicited CFTR-mediated currents (I*_SC_*) in F508del/F508del human bronchial epithelial (HBE) cells chronically treated with the peptide (Supplemental Figure 2, A and B), indicating a lack of functional CFTR channels at the cell surface. In contrast, cells treated with the CFTR corrector VX-809 showed robust CPTcAMP/VX-770–stimulated currents (Supplemental Figure 2, A and B). These results confirm that PI3Kγ MP does not enhance F508del-CFTR maturation.

### PI3Kγ MP activates PKD1.

**Figure 3:**
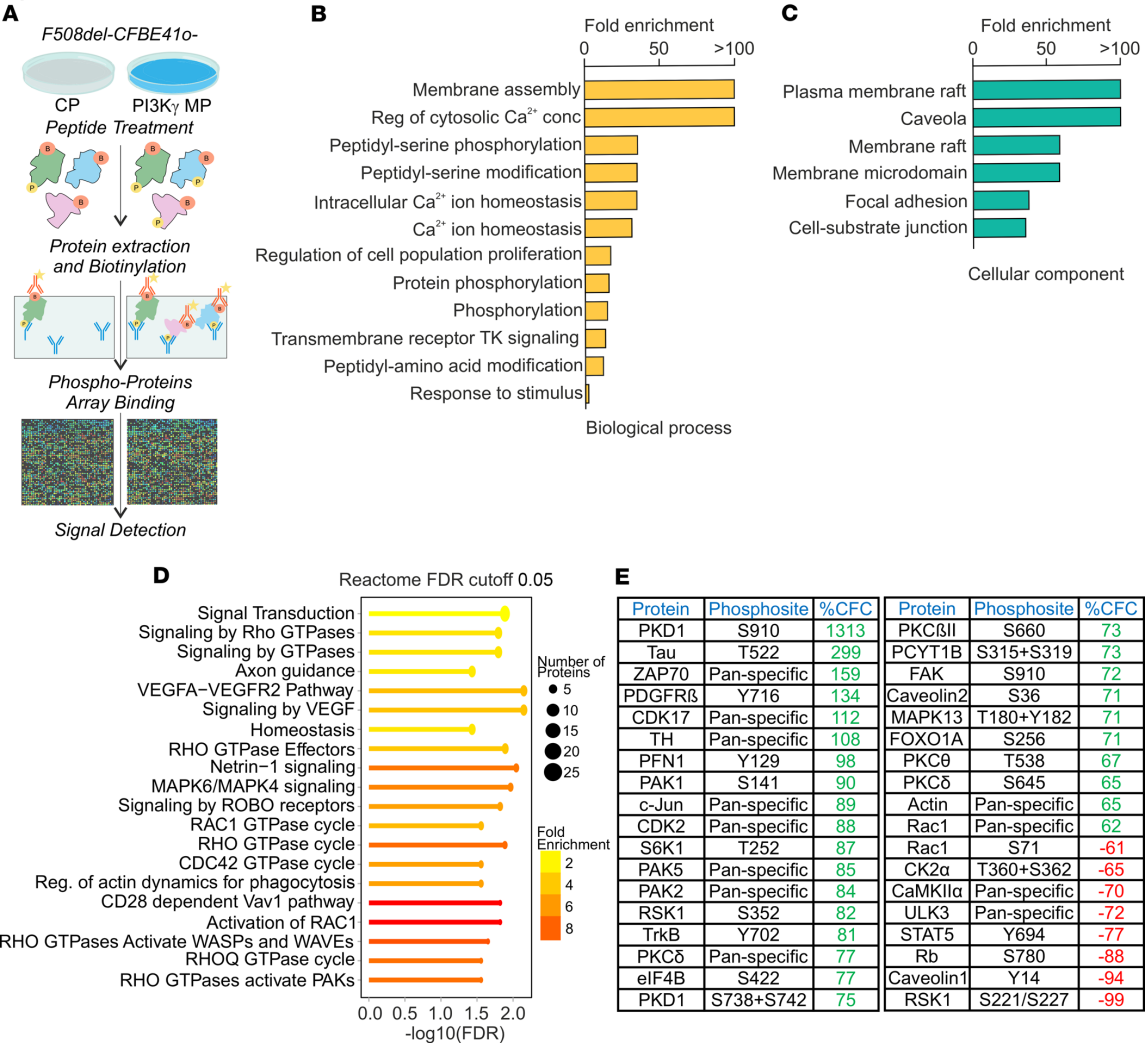
Phosphoproteomic analysis reveals that PI3Kγ MP modulates proteins involved in membrane remodeling and trafficking. (**A**) Experimental workflow used for phosphoproteomic analysis of F508del-CFBE41o- cells treated with control peptide (CP) or PI3Kγ MP (25 μM, 30 minutes). A phospho-specific microarray, containing 875 phosphosite-specific and 451 pan-specific antibodies, was used. Thirty-six proteins showing phosphorylation changes exceeding ±60% compared with the control (expressed as percentage CFC, i.e., percentage fold-change compared with control) were selected for downstream analysis. (**B** and **C**) Panther Gene Ontology (GO) slim-term enrichment analysis of proteins with altered phosphorylation after PI3Kγ MP treatment. Significantly enriched GO terms (FDR < 0.05) were categorized under (**B**) biological processes and (**C**) cellular components. (**D**) Reactome pathway enrichment analysis of differentially phosphorylated proteins after PI3Kγ MP treatment. Lines represent the top 20 pathways; *x* axis shows the –log_10_(FDR). Color intensity reflects fold enrichment, and circle size indicates the number of proteins; color intensity (yellow to red) indicates increasing fold enrichment. The full phospho-array protein list served as background reference. (**E**) Proteins with CFC greater than 60% (green) or less than −60% (red). A CFC of 100% corresponds to a 2-fold increase in signal intensity after PI3Kγ MP treatment relative to CP.

**Figure 4:**
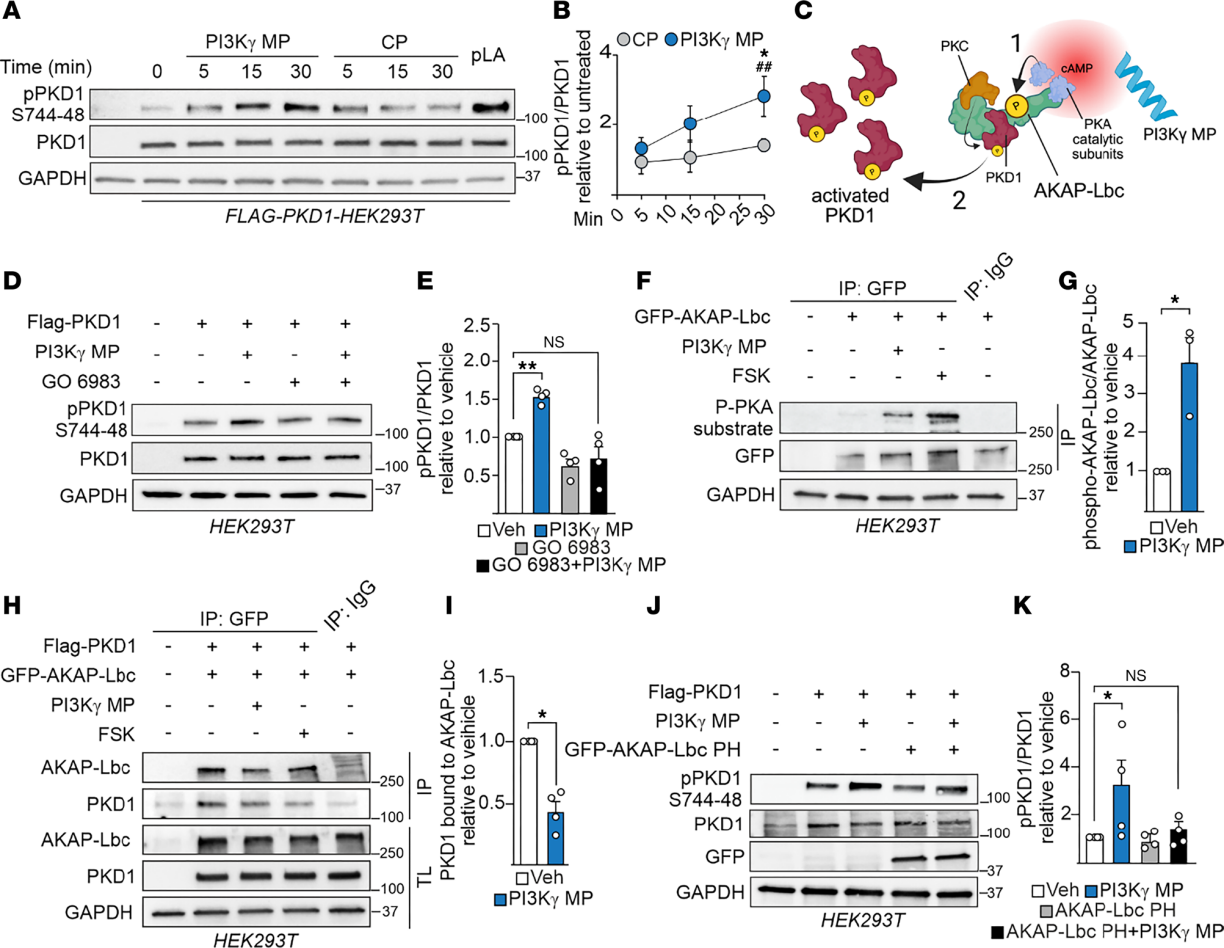
PI3Kγ MP activates AKAP-Lbc–anchored PKD1. (**A** and **B**) Representative Western blot (**A**) and relative quantification (**B**) of phospho-PKD1 (pPKD1 S744-748) in HEK293T cells overexpressing PKD1 and treated with CP or PI3Kγ MP (25 μM). Poly-L-arginine (5 μM, 10 minutes) served as positive control. *n* = 3. (**C**) Proposed mechanism of PKD1 activation: PI3Kγ MP elevates cAMP levels, leading to PKA activation, phosphorylation of AKAP-Lbc, and full activation and release of PKD1 from signalosome. (**D** and **E**) Representative Western blot (**D**) and relative quantification (**E**) of pPKD1 S744-748 in HEK293T cells overexpressing FLAG-PKD1 and treated with PI3Kγ MP (25 μM, 30 minutes) ± PKC inhibitor GO6983 (0.5 μM, 30 min preincubation). *n* = 4. (**F** and **G**) Representative Western blot (**F**) and relative quantification (**G**) of phosphorylated AKAP-Lbc in HEK293T cells overexpressing GFP-AKAP-Lbc and treated with vehicle, PI3Kγ MP (25 μM, 30 minutes), or Fsk (1.5 μM, 10 minutes; positive control). AKAP-Lbc was immunoprecipitated and IP pellets probed with PKA substrate antibody. TL, total lysate. *n* = 3. (**H** and **I**) Representative Western blot (**H**) and relative quantification (**I**) of PKD1 bound to AKAP-Lbc in HEK293T cells overexpressing GFP-AKAP-Lbc and FLAG-PKD1, treated with vehicle, PI3Kγ MP, or forskolin as in **F**. *n* = 3. (**J** and **K**) Representative Western blot (**J**) and relative quantification (**K**) of pPKD1 S744-748 in HEK293T cells overexpressing FLAG-PKD1 alone or with AKAP-Lbc PH domain, treated with vehicle or PI3Kγ MP (25 μM, 30 minutes). *n* = 4. In **B**, ^##^*P* < 0.01 for PI3Kγ MP at t = 30 versus t = 0 minutes; *P < 0.05 PI3Kγ MP versus CP at t = 30 minutes by 2-tailed Student’s *t* test. In **G** and **I**, *P < 0.05 PI3Kγ MP versus vehicle by Student’s *t* test. In **E** and **K**, *P < 0.05, **P < 0.01 by 1-way ANOVA with Dunnett’s multiple-comparison test. Data shown as mean ± SEM; *n* = independent experiments; data points = independent biological replicates.

To gain further mechanistic insight into the PI3Kγ MP action, a targeted phosphoproteomics approach was performed in F508del-CFBE41o- cells (Figure 3A), with the aim of identifying kinases and regulatory proteins modulated by the peptide. PI3Kγ MP induced phosphorylation changes in a subset of proteins implicated in membrane remodeling and calcium (Ca^2+^) homeostasis (Figure 3, B and C) as well as protein trafficking (Figure 3D), like small GTPases from the Rho/Rac/Cdc42 families (16). Notably, PKD1, a well-established regulator of Golgi-to–plasma membrane trafficking, endocytosis, and recycling of membrane proteins (17), exhibited the most significant relative increase in phosphorylation in cells treated with PI3Kγ MP compared with those exposed to CP (Figure 3E). Phosphoproteomics results were confirmed by Western blot assays showing that PI3Kγ MP but not CP triggered PKD1 phosphorylation on activating serines 744 and 748 in HEK293T cells overexpressing the kinase (Figure 4, A and B).

Next, we aimed to investigate how the increase in cAMP triggered by PI3Kγ MP could lead to PKD1 activation. Previous work demonstrated that PKD1 activation requires the coordinated action of PKC and PKA, which is facilitated by AKAP-Lbc. Within the AKAP-Lbc complex, PKC phosphorylates PKD1 at serines 744 and 748, and PKA-mediated phosphorylation of the AKAP promotes the release of the activated kinase (18) (Figure 4C). Pharmacological PKC inhibition with GO6983 significantly reduced the PKD1 phosphorylation on serines 744 and 748 elicited by PI3Kγ MP (Figure 4, D and E), indicating that the ability of the peptide to trigger PKD1 depends on PKC. Furthermore, PI3Kγ MP increased PKA-mediated phosphorylation of AKAP-Lbc by 4-fold (Figure 4, F and G), which corresponded with an approximately 50% reduction in binding with PKD1 in HEK293T cells coexpressing GFP-AKAP-Lbc and PKD1 (Figure 4, H and I). To further demonstrate that PI3Kγ MP activates an AKAP-Lbc–anchored pool of PKD1, HEK293T cells were transfected with a plasmid encoding for a soluble version of the pleckstrin homology (PH) domain of AKAP-Lbc, which antagonizes PKD1 activation by displacing PKC from the complex (18). In these cells, PI3Kγ MP failed to significantly raise PKD1 phosphorylation over basal values (Figure 4, J and K), demonstrating that the peptide controls a pool of PKD1 tethered to AKAP-Lbc. Overall, these data support a model in which PI3Kγ MP–induced cAMP elevation activates a localized pool of PKA, which in turn mediates full PKD1 activation and its subsequent release from the AKAP-Lbc signalosome.

### PKD1 activation by PI3Kγ MP enhances F508del-CFTR membrane localization, maximizing the efficacy of ETI therapy.

**Figure 5:**
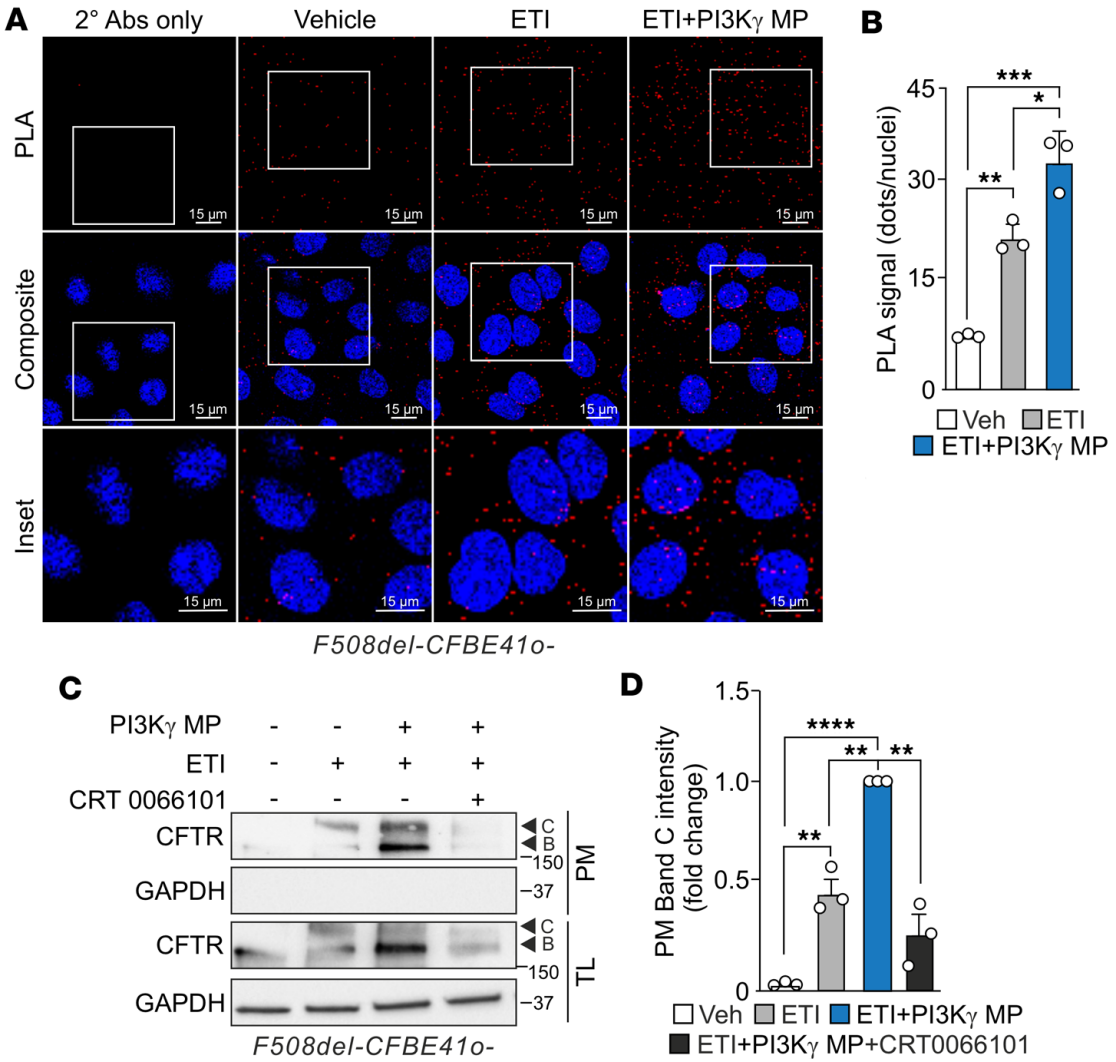
PKD1 activation by PI3Kγ MP enhances F508del-CFTR membrane localization. (**A**) Representative images showing red proximity ligation assay (PLA) signals indicating the interaction between F508del-CFTR and PKD1 in F508del-CFBE41o- cells. Cells were treated with ETI (3 μM VX-445, 10 μM VX-661, and 1 μM VX-770), alone or with PI3Kγ MP (25 μM) for 24 hours. Nuclei were stained with DAPI (blue). Scale bar: 15 μm. (**B**) Quantification of PLA dots per cell as in **A**. *P < 0.05, **P < 0.01, ***P < 0.001 by 1-way ANOVA with Bonferroni’s post hoc correction. *n* = 3. (**C**) Representative Western blot of CFTR and GAPDH in plasma membrane fractions and total lysates (TLs) of F508del-CFBE41o- cells treated with ETI, PI3Kγ MP (25 μM), or the PKD inhibitor CRT0066101 (10 μM) for 24 hours. GAPDH was absent in plasma membrane fractions and used as a loading control in TLs. (**D**) Quantification of plasma membrane CFTR band C intensity as shown in **C**, expressed as fold-change relative to ETI + PI3Kγ MP. **P < 0.01, ****P < 0.0001 by 1-way ANOVA with Bonferroni’s post hoc test. *n* = 3. Data are shown as mean ± SEM. *n*: number of independent experiments. Data points represent independent biological replicates.

Next, we evaluated whether the pool of PKD1 activated by the peptide and released from the AKAP-Lbc signalosome localizes to compartments that contain CFTR, potentially influencing its plasma membrane localization. To this end, we tested the association between PKD1 and CFTR by proximity ligation assay (PLA), a test detecting proteins in close vicinity (<40 nm). Our results showed that in CFBE-F508del-CFBE41o- cells under ETI therapy, the number of PLA puncta significantly increased upon treatment with PI3Kγ MP (Figure 5, A and B). To assess the extent to which PI3Kγ MP-activated PKD1 contributes to increased plasma membrane expression of F508del-CFTR, we inhibited PKD1 using the inhibitor CRT0066101. PKD1 inhibition completely abolished the increase in F508del-CFTR plasma membrane density elicited by PI3Kγ MP in CFBE-F508del-CFBE41o- cells treated with ETI (Figure 5, C and D), demonstrating that the ability of PI3Kγ MP to maximize the effect of ETI on F508del-CFTR plasma membrane localization requires PKD1.

**Figure 6:**
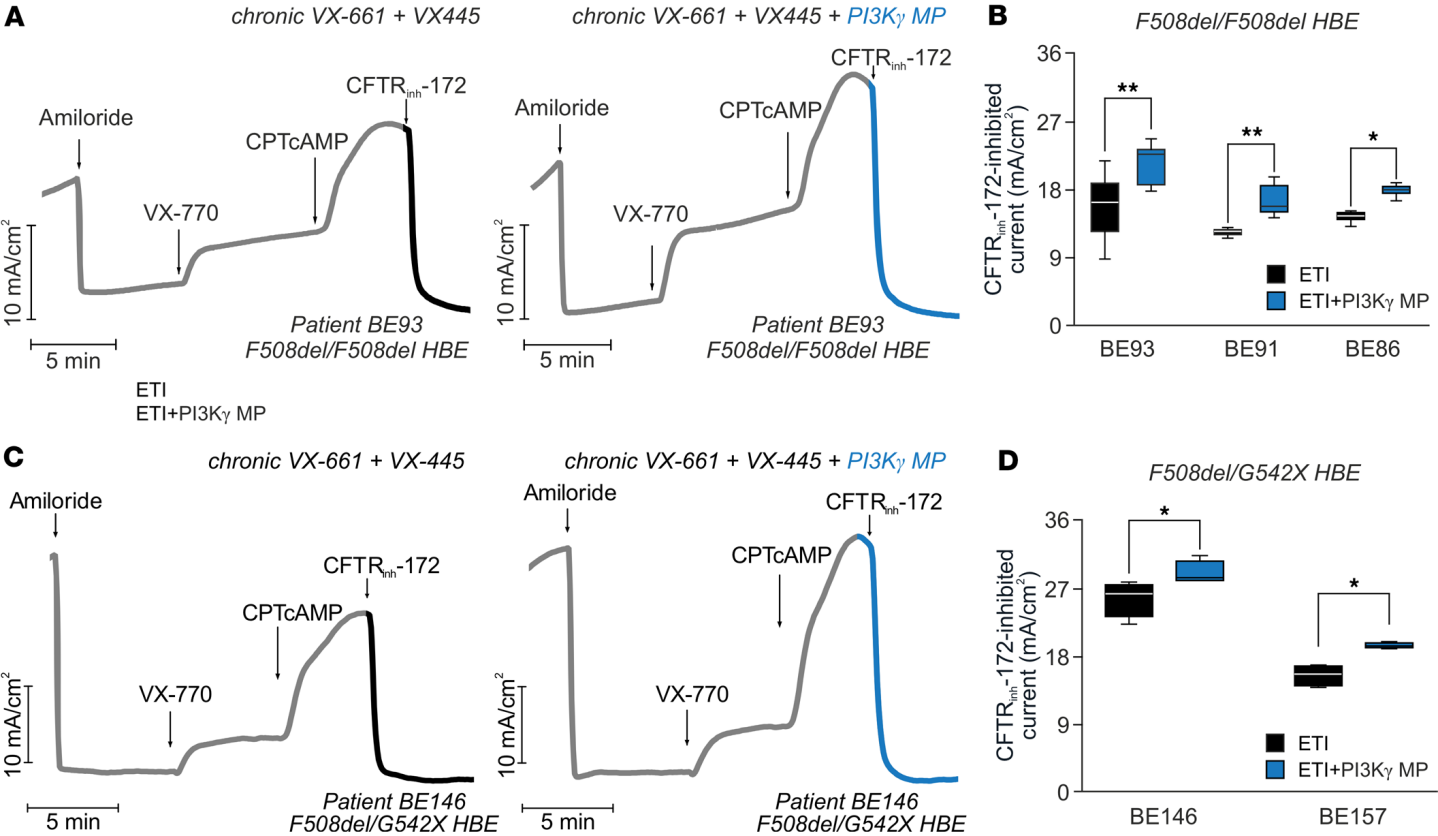
PI3Kγ MP enhances ETI efficacy in human bronchial epithelial cells from individuals with CF. (**A**) Representative short-circuit current (I*_SC_*) traces in primary human bronchial epithelial (HBE) cells from a F508del/F508del CF donor (patient BE93) grown at the air-liquid interface (ALI). Cells were corrected for 24 hours with 10 μM VX-661 and 3 μM VX-445, alone or with PI3Kγ MP (10 μM), and then acutely exposed to amiloride (100 μM), VX-770 (1 μM), CPTcAMP (100 μM), and CFTR_inh_-172 (10 μM). (**B**) Average current inhibition by CFTR_inh-_172 (10 μM) in primary HBE cells from 3 F508del/F508del donors. For BE93, *n* = 12 (ETI) and *n* = 9 (ETI + PI3Kγ MP) technical replicates. For BE91 and BE86, *n* = 5 and *n* = 3 technical replicates per treatment, respectively. *P < 0.05, **P < 0.01 by 2-tailed Student’s *t* test. (**C**) Representative I*_SC_* traces in primary HBE cells from a F508del/G542X CF donor (patient BE146) grown at the ALI. Cells were corrected for 24 hours with VX-661 (10 μM) and VX-445 (3 μM), alone or with PI3Kγ MP (10 μM), followed by acute application of amiloride (100 μM), VX-770 (1 μM), CPTcAMP (100 μM), and CFTR_inh_-172 (10 μM) at the indicated times. (**D**) Average current inhibition by CFTR_inh_-172 (10 μM) in HBE cells from 2 F508del/G542X donors (BE146 and BE157); *n* = 4 technical replicates each. *P < 0.05 by Mann-Whitney *U* test. Data are shown as mean ± SEM.

Finally, we aimed to assess the functional impact of this regulatory mechanism. To this end, we measured CFTR-dependent Cl^–^ secretion in F508del/F508del primary human bronchial epithelial (HBE) cells treated with ETI alone or combined with PI3Kγ MP. The membrane-permeable cAMP analog CPTcAMP was used to induce maximal CFTR activation, which is directly proportional to the channel plasma membrane density. In F508del/F508del HBE cells from 3 independent donors, the maximal CFTR activity elicited by CPTcAMP, and the relative current drop induced by the specific CFTR inhibitor CFTR_inh_-172 at the end of the experiment, were 30% higher in cells co-treated with PI3Kγ MP than in those exposed to ETI alone (Figure 6, A and B, and Supplemental Figure 3, A and B). Similar results were obtained in HBE cells derived from 2 patients with a more severe genotype, carrying the F508del mutation and the nonsense G542X mutation (Figure 6, C and D, and Supplemental Figure 3C). Overall, these results demonstrate that PI3Kγ MP maximizes the efficacy of the triple-combination ETI by increasing the plasma membrane density of F508del channels through the activation of PKD1.

## Discussion

Our findings revealed a regulatory mechanism governing CFTR membrane localization under the control of the AKAP function of PI3Kγ. Furthermore, our results support the pharmacological targeting of this pathway using PI3Kγ MP (10) as a promising strategy to overcome the limitations of current CFTR modulator therapies.

The triple-combination therapy ETI, the standard of care for patients with CF, reinstates only partially the activity of F508del-CFTR, up to a maximum of 50% of WT CFTR activity (5). This limitation arises from the inability of correctors to fully stabilize the mutant channel and restore its trafficking to the plasma membrane (19). Our findings not only support this notion but also propose a potential solution. We demonstrate that the physiological and compartment-restricted increase in cAMP achieved with PI3Kγ MP (10) enhances the ability of ETI to augment the surface expression of F508del-CFTR. Importantly, this effect is not due to the peptide acting as a CFTR corrector itself since, in the absence of pharmacological chaperones that rescue folding and ER-to-Golgi trafficking defects, the peptide fails to increase the levels of mature, fully glycosylated CFTR at the plasma membrane. Our data support a model in which CFTR correctors are necessary to enable the mutant channel to pass the ER quality control, thereby allowing PI3Kγ MP to exert its effect. This underscores the therapeutic importance of combining PI3Kγ MP with CFTR correctors. In the absence of correction, the misfolded channel accumulates in the ER and can trigger an alternative, unconventional secretory pathway that delivers immature CFTR to the plasma membrane (20). While our data suggest that the peptide may influence this pathway, this does not result in increased levels of functional CFTR, further reinforcing the need to pair PI3Kγ MP with correctors to achieve meaningful therapeutic outcomes.

Building on earlier studies demonstrating the potential of cAMP to enhance the efficacy of CFTR correctors (11, 12), our work extends this concept by identifying PKD1 as a critical downstream effector that links cAMP signaling to CFTR membrane localization. PKD1 is a well-established regulator of protein trafficking and secretion. Prior research has shown that PKD1 activation facilitates Golgi-to-plasma membrane transport of several cargoes, including the glucose transporter GLUT4, insulin, and various interleukins (21–23). Moreover, PKD1 has been reported to increase plasma membrane levels of αvβ3 and α5β1 integrins by inhibiting their endocytosis and promoting their recycling (24, 25). Our study broadens the functional repertoire of PKD1 by identifying its role in the regulation of CFTR biogenesis. We demonstrate that PKD1 localizes within CFTR-positive compartments, and that its pharmacological inhibition reduces the plasma membrane density of F508del-CFTR. Given the established involvement of PKD1 in multiple stages of the secretory and recycling pathways, the increased surface expression of F508del-CFTR observed upon PI3Kγ MP treatment is consistent with a combination of enhanced forward trafficking, decreased endocytosis, and improved recycling, all of which may be regulated by PKD1. Clarifying the relative contribution of these mechanisms to CFTR maturation and surface stability remains an important objective, but a detailed dissection of PKD1 function along the trafficking itinerary lies beyond the scope of this initial study.

Although PKD1 emerged as the most prominently hyperphosphorylated target in our phosphoproteomic analysis, other proteins previously implicated in cytoskeleton regulation and CFTR stabilization at the plasma membrane, such as PAKs, Rac1, FAK, and actin itself (11, 26–29), were also significantly hyperphosphorylated in PI3Kγ MP–treated cells. These findings suggest that PKD1 may intersect with these pathways to modulate the actin network, ultimately influencing CFTR trafficking and retention. However, additional studies will be required to more precisely delineate the mechanistic relationship between PKD1 activation and cytoskeletal remodeling. Nonetheless, our mechanistic analysis revealed that, in response to PI3Kγ MP-induced cAMP elevation, PKD1 activation depends on its close spatial proximity to a localized pool of the cAMP-dependent kinase PKA. This organization is mediated by AKAP-Lbc, a scaffold protein that anchors both kinases within the same signaling module and has also been implicated in the regulation of protein secretion (30). Notably, AKAP-Lbc assembles a functional signaling complex that includes not only PKA but also PKC, both of which are essential for full PKD1 activation. While PKC directly phosphorylates PKD1, PKA phosphorylates AKAP-Lbc itself, an event required for the release of activated PKD1 from the scaffold (18). Our observation that PKC inhibition prevents PI3Kγ MP–induced PKD1 activation suggests that the peptide influences both the PKA- and PKC-dependent pathways leading to PKD1 activation. This is consistent with our previous findings that PI3Kγ MP modulates intracellular Ca^2+^ levels and activates Ca^2+^-dependent channels (10), a pathway known to stimulate PKC. This model is further supported by our phosphoproteomic data, which reveal changes in the phosphorylation of multiple PKC isoforms after PI3Kγ MP treatment and are consistent with the well-established role of PKC in regulating CFTR activity (31). Furthermore, our data showing enhanced AKAP-Lbc phosphorylation and reduced PKD1 binding upon peptide treatment support the hypothesis that PKA engagement by PI3Kγ MP is critical for releasing activated PKD1 from the AKAP-Lbc complex. This release likely enables PKD1 to traffic to CFTR-positive compartments, where it contributes to the regulation of CFTR membrane localization.

Our results demonstrate that this mechanism regulating CFTR membrane abundance holds promising therapeutic potential. Short-circuit current measurements in primary HBE cells from patients with CF harboring mutations currently eligible for ETI therapy reveal that treatment with PI3Kγ MP significantly increases the amount of plasma membrane–localized F508del-CFTR available for activation by the cAMP analog, CPTcAMP. This results in an approximately 30% increase in maximal CFTR activity compared with ETI therapy alone. This observation adds to our previous finding that, by directly affecting CFTR Ser-737 phosphorylation, PI3Kγ MP heightens VX-770–mediated gating of the channel (10). Thus, the ability of PI3Kγ MP to potentiate CFTR modulator efficacy arises from its dual action on both membrane localization and the gating mechanisms of CFTR. In addition to demonstrating that the localized cAMP elevation induced by PI3Kγ MP promotes not only PKA-dependent CFTR gating (10) but also its membrane localization, the current study extends our previous findings by broadening the clinical relevance of PI3Kγ MP to patient genotypes beyond F508del homozygotes. Although the effect of PI3Kγ MP was validated across 5 CF donors carrying either F508del/F508del or F508del/G542X genotypes, we acknowledge that larger patient cohorts will be required in future studies to more precisely define the magnitude of clinical benefit. These analyses will be possible as the compound moves through clinical development (32).

In summary, our results identify a regulatory pathway governing CFTR membrane expression, which may be exploited to overcome the limitations of current CFTR modulator therapies. Pharmacological engagement of this mechanism with a peptide that targets the scaffolding function of PI3Kγ activates a cAMP-dependent signalosome involving AKAP-Lbc, PKC, and PKD1, ultimately enhancing membrane localization of F508del-CFTR in combination with ETI therapy. This mechanism of action offers key advantages over strategies that induce broad cAMP elevation, such as the cell-permeable, non-hydrolysable analog CPTcAMP and, importantly, clinically used PDE4 inhibitors. The clinical benefit of PDE4 inhibitors is often hindered by adverse effects arising from indiscriminate inhibition of all PDE4 isoforms, including those not directly involved in CFTR regulation, as well as from systemic PDE4 blockade. Our previous proof-of-concept work, together with the present study, demonstrate that PI3Kγ MP overcomes these limitations. By selectively targeting PI3Kγ-dependent PDE4 isoforms, PI3Kγ MP produces a spatially confined cAMP response that specifically affects the CFTR lifecycle. Moreover, PI3Kγ MP shows favorable airway-restricted bioavailability and minimal systemic exposure after aerosol delivery, which maximize its therapeutic index (10). Notably, PI3Kγ MP is a clinically advanced compound, having completed preclinical safety and toxicology studies in animal models and a phase I clinical trial evaluating the safety and tolerability of single and multiple doses in healthy volunteers. The peptide is now being developed as a CFTR enhancer, intended as an add-on therapy to existing CFTR modulators in patients with CF (32). Collectively, these findings provide a strong foundation for the accelerated translational development of PI3Kγ MP as an inhalable adjunct therapy designed to enhance the long-term effectiveness of CFTR modulators in individuals with CF.

## Methods

### Sex as a biological variable

This study used HBE cells derived from 5 individuals with CF. The sex of the donors was not always disclosed (based on the informed consent), but this variable was not considered during sample selection, and sex-specific analyses were not performed. Given the focus on cell-autonomous mechanisms of CFTR regulation, the findings are expected to be relevant regardless of donor sex.

### Antibodies, plasmids, shRNA, peptides, and reagents

#### Antibodies.

The anti-CFTR antibody A2-570 was provided by J.R. Riordan (University of North Carolina at Chapel Hill) via the CFTR Antibody Distribution Program of the Cystic Fibrosis Foundation. The anti–phospho-Ser/Thr-PKA substrate (catalog 9621S), anti-PKD/PKCμ (catalog 90039), anti-tubulin (catalog 2144), and anti-vinculin (catalog 4650) antibodies were purchased from Cell Signaling Technology. The anti–phospho-Ser910 of PKCμ (catalog PA538388) and anti–Ser744, Ser748 phospho-PDK/PKCμ (catalog PA5110148) antibodies were purchased from Invitrogen. The anti-GAPDH (catalog sc-3223) antibody was purchased from Santa Cruz Biotechnology. The anti-FLAG M2 (F1804), anti-GFP (G1544), anti-mouse IgG (catalog 12-371), and anti-rabbit IgG (catalog 12-370) antibodies were purchased from Sigma-Aldrich.

#### Drugs.

Elexacaftor (VX-445), tezacaftor (VX-661), ivacaftor (VX-770), and Go 6983were purchased from MedChemExpress. Lumacaftor (VX-809) was purchased from Selleck Chemicals. DMSO, CRT0066101 hydrochloride, CHX solution (C4859), poly-L-arginine hydrochloride (P4663), forskolin, amiloride, CPTcAMP, and CFTR_inh_-172 were purchased from Merck.

#### Other reagents.

Endo H was purchased from New England Biolabs. Pierce high-capacity streptavidin agarose and EZ-Link sulfo-NHS-SS-biotin were purchased from Thermo Fisher Scientific. Penentratin-1 (CP: RQIKIWFQNRRMKWKK), and the PI3Kγ-mimetic peptide (PI3Kγ MP: RQIKIWFQNRRMKWKKGKATHRSPGQIHLVQRHPPSEESQAF) were synthesized by GenScript.

#### Plasmids.

The pEGFP-C1 plasmids expressing WT-CFTR-GFP and the F508del mutant CFTR (F508del-CFTR-GFP) were gifts from P. Haggie (UCSF). Flag-PKD1 plasmid was purchased from VectorBuilder. The GFP-AKAP-Lbc and the GFP-AKAP-Lbc PH domain plasmids were described previously (18).

### Cell lines and transfection

Immortalized CF HBE (CFBE41o-) cells stably expressing the F508del-CFTR (F508del-CFBE41o-) were provided by L. Fu from the UAB Research Foundation. Cells were cultured in MEM (21090022; Gibco) supplemented with 10% FBS (10270106; Gibco), 2% L-glutamine (25030024; Gibco), and 100 U/mL penicillin and 100 μg/mL streptomycin (15140122; Gibco), with 3 μg/μL puromycin (A1113803; Gibco) on culture dishes precoated with 1 mg/mL human fibronectin (F0895; Sigma-Aldrich), 3 mg/mL bovine collagen I (C4243; Sigma-Aldrich), and 0.1% BSA (A9418; Sigma-Aldrich). HEK293T cells (American Type Culture Collection; CRL-3216) were cultured in DMEM (61965026; Gibco) supplemented with 10% FBS (10270106; Gibco) and 100 U/mL penicillin and 100 μg/mL streptomycin (15140122; Gibco). Cells were transfected with the indicated plasmids (up to 10 μg of total cDNA) with either calcium phosphate or X-tremeGENE HP DNA transfection reagent (6366244001; Roche) and used for experiments 24 hours after transfection. All cells were cultured at 37°C and under a 5% CO_2_ atmosphere.

### Protein extraction and IP

CFBE41o- and HEK293T cells were scraped in 120 mmol/L NaCl, 50 mmol/L Tris-HCl (pH 8.0), and 1% Triton X-100, supplemented with proteases (Roche) and phosphatase inhibitors (10 mM Na_4_P_2_O_7_; 10 mM NaF; 2 mM Na_3_VO_4_), as described above. Detergent-insoluble material was precipitated by centrifugation at 16,200*g* for 10 minutes at 4°C. Supernatants were subjected to IP or used directly for Western blotting. For IP assays, precleared extracts were incubated with 30 μL of a 2:1 slurry of protein G-sepharose (Amersham Biosciences) and 1–2 μg of antibody/mg of protein for 2 hours at 4°C. Immunocomplexes were then extensively washed with lysis buffer and used for Western blotting.

### Cell surface biotinylation assay

Cells were washed twice with ice-cold PBS (pH 8.0) and incubated with 0.5 mg/mL sulfo-NHS-SS-biotin (Thermo Fisher Scientific) in PBS (pH 8.0) for 1 hour at 4°C under gentle agitation. Cells were washed once with 25 mM Tris (pH 8.0) to quench the non-reacted biotinylation reagent and twice with ice-cold PBS to remove the non-reacted biotinylation reagent. Protein extraction was performed as described above. An aliquot of supernatants corresponding to 600 μg of proteins was incubated with high-capacity streptavidin agarose resin (Thermo Fisher Scientific) for 2 hours at 4°C with gentle mixing, following the manufacturer’s recommendation. Streptavidin-bound complexes were pelleted at 1,000*g* and washed 4 times with lysis buffer. Biotinylated proteins were eluted from the resin with reducing sample buffer twice and directly used for Western blotting.

### Endo H assay

Digestion of glycosylated CFTR by Endo H (New England Biolabs) was performed according to the manufacturer’s protocol. Briefly, surface biotinylated and avidin-bound proteins or total protein extracts were incubated with a denaturing buffer (0.5% SDS and 0.4 M DTT) in a total reaction volume of 20 μL and incubated at 37°C for 10 minutes. Samples were then treated with Endo H (1,000 U/reaction) in GlycoBuffer (0.05 M sodium citrate, pH 5.5) at 37°C for 1 hour. Digested samples were then mixed with 2× Laemmli sample buffer before Western blot analysis.

### Phosphoproteomics

After treatment with 25 μM PI3Kγ MP or an equimolar amount of CP for 30 minutes, F508del-CFBE41o- cells were lysed as described above, and protein samples were frozen at –80°C before being subjected to an antibody microarray (KAM-1325 array) and data analysis, which was performed at Kinexus. The array, which monitors changes in the expression levels and phosphorylation states of signaling proteins, includes 875 phosphosite-specific antibodies (for phosphorylation) and 451 pan-specific antibodies (for expression levels of these phosphoproteins). The resultant changes were expressed as a percentage of change to the control (% CFC). Only targets with CFC above 60% or below –60% were considered real significant changes.

### Western blotting

Protein concentrations in cell extracts were measured using the Bio-Rad protein assay system. Proteins were separated on 4%–20% gradient SDS-PAGE gels and transferred onto PVDF membranes. Membranes were blocked for 1 hour at room temperature (RT) with 5% BSA in Tris-buffered saline containing 0.3% Tween 20 (TBST). Primary antibodies, diluted in TBST at the specified ratios, were applied to the membranes and incubated overnight at 4°C. Detection of primary antibody binding was performed using HRP-conjugated secondary antibodies (Sigma-Aldrich) and developed with a chemiluminescence ECL assay kit (Millipore Corporation). Protein band quantification was carried out using Quantity One analysis software (Bio-Rad).

### Immunogold electron microscopy

Cells were plated on Alcian blue–coated glass coverslips and fixed for 10 minutes with 0.05% glutaraldehyde in 4% paraformaldehyde (PFA) electron microscopy (EM) grade and 0.2 M HEPES buffer, and 50 minutes in 4% PFA EM grade in 0.2 M HEPES buffer. After 3 washes in PBS, cells were incubated for 10 minutes with 50 mM glycine and blocked for 1 hour in blocking buffer (0.2% BSA, 5% goat serum, 50 mM NH_4_Cl, 0.1% saponin, 20 mM PO_4_ buffer, 150 mM NaCl). Staining with primary antibodies and nanogold-labeled secondary antibodies (Nanoprobes) was performed in blocking buffer at RT. Cells were fixed for 30 minutes in 1% glutaraldehyde and the nanogold was enlarged with gold enhancement solution (Nanoprobes) according to the manufacturer’s instructions. Cells were post-fixed with osmium tetroxide, embedded in EPON resin, and processed into ultrathin slices. After contrasting with uranyl acetate and lead citrate, the sections were analyzed with a transmission electron microscope, Talos L120C (FEI, Thermo Fisher Scientific), operating at 120 kV. Images were acquired with a Ceta CCD camera.

### PLA

The Duolink in situ red starter kit mouse/rabbit (Sigma-Aldrich) was used according to the manufacturer’s protocol for the PLA. Briefly, F508del-CFBE41o- cells were seeded (30,000 cells/well) in a 24-well plate containing 12 mm sterile and coated coverslips. After 24 hours, cells were treated for 24 hours with 3 μM elexacaftor (VX-445), 10 μM tezacaftor (VX-661), and 1 μM ivacaftor (VX-770) alone or together with PI3Kγ MP (25 μM). Cells were then washed once with 1× PBS and fixed in 4% paraformaldehyde for 20 minutes at RT. Next, cells were washed 3 times with 1× PBS and permeabilized with 0.1% Triton X-100 in 1% BSA/PBS for 20 minutes at RT. Blocking was performed with the Duolink blocking solution for 1 hour at 37°C. Primary antibodies were diluted in antibody diluent, added, and incubated overnight at 4°C. The following day, cells were washed twice with wash 1× buffer A at RT for 5 minutes each, and subsequently incubated with the Duolink PLUS and MINUS probes (1:5 diluted in antibody diluents) for 1 hour at 37°C. After 2 additional washes with wash 1× buffer A, ligation of the probes was conducted by incubating the cells with a ligase for 30 minutes at 37°C. Before the amplification step, cells were washed twice with wash 1× buffer A and then incubated with a polymerase for 100 minutes at 37°C. After 2 final washes with wash 1× buffer B and 1 wash with wash 0.01× buffer B, coverslips were mounted onto slides with Duolink in situ mounting media with DAPI for 15 minutes at RT, and then stored at –20°C before acquisition. Images were acquired using a Leica TCS SP5 confocal laser scanning microscope with a 40× objective.

### Primary HBE cell culture

Experiments with primary HBE cells were conducted at the Istituto Giannina Gaslini following a previously established protocol (10). In brief, epithelial cells were isolated from the mainstem bronchi of patients with CF undergoing lung transplantation. Cells were derived from 2 donors homozygous for the F508del mutation and 2 with the F508del/G542X genotype. Bronchial tissues were incubated overnight at 4°C in a protease XIV–containing solution to isolate epithelial cells. Cells were cultured in a serum-free medium (1:1 mix of LHC9 and RPMI 1640) enriched with hormones and supplements to promote cell proliferation. Of note, the culture medium initially included a combination of antibiotics (typically colistin, piperacillin, and tazobactam) to eliminate bacterial contamination. To induce epithelial differentiation, cells were seeded at a high density (500,000 cells per 1 cm^2^) on porous Snapwell inserts (Corning). After 24 hours, the serum-free medium was replaced with DMEM/Ham’s F12 supplemented with 2% FBS and additional growth factors. Differentiation into a polarized tight epithelium was assessed by measuring transepithelial electrical resistance and potential difference using an epithelial voltohmmeter (EVOM1; World Precision Instruments). The culture medium was refreshed daily on both sides of the membrane for 8 to 10 days (liquid-liquid interface). Subsequently, the apical medium was removed, transitioning the cells to an air-liquid interface to enhance epithelial maturation. Cells were maintained under air-liquid interface conditions for 2 to 3 weeks before experimental use.

### Short-circuit current measurements

Short-circuit current (I*_SC_*) measurements in primary HBE cells were performed at the Istituto Giannina Gaslini, following a previously established protocol (10). Differentiated bronchial epithelia cultured on Snapwell inserts were mounted in vertical diffusion chambers resembling Ussing chambers with internal fluid circulation. Both the apical and basolateral hemi-chambers were filled with 5 mL of a solution containing 126 mM NaCl, 0.38 mM KH_2_PO_4_, 2.13 mM K_2_HPO_4_, 1 mM MgSO_4_, 1 mM CaCl_2_, 24 mM NaHCO_3_, and 10 mM glucose. The chambers were continuously bubbled with a 5% CO_2_/95% air gas mixture, and the temperature was maintained at 37°C. A voltage clamp (DVC-1000; World Precision Instruments) was used to short-circuit the transepithelial voltage, with connections to the apical and basolateral chambers via Ag/AgCl electrodes and agar bridges (1 M KCl in 1% agar). Voltage offsets and fluid electrical resistance were compensated before each experiment. The I*_SC_* signals were recorded using a PowerLab 4/25 analog-to-digital converter (ADInstruments) connected to a computer.

### Statistics

Raw data were first analyzed to confirm their normal distribution via the Shapiro-Wilk test and then analyzed by unpaired Student’s *t* test or 1-way ANOVA. Welch’s *t* test was used as appropriate, depending on equality of variances. Bonferroni’s or Dunnett’s correction (1-way ANOVA) was applied to correct for multiple comparisons, as appropriate. In the absence of a normal distribution, nonparametric Kruskal-Wallis or Mann-Whitney *U* tests were used, followed by Dunn’s correction for multiple comparisons if appropriate. A *P* value less than 0.05 was considered significant.

### Study approval

The collection of bronchial epithelial cells and their study to investigate the mechanisms of transepithelial ion transport and the response to CFTR modulators were specifically approved by the Ethics Committee of the Istituto Giannina Gaslini following the guidelines of the Italian Ministry of Health. Each patient provided written informed consent to the study using a form that was also approved by the Ethics Committee.

### Data availability

All data supporting the findings of this study are available within the main text, the supplemental materials, and the Supporting Data Values file. Phosphoproteomic data generated using the Kinexus platform are provided as Supplemental Dataset 1 (Kinexus_KAM-1325 array.xlsx). PI3Kγ MP is available to the scientific community upon completion of a material transfer agreement with Kither Biotech. The following cell lines and reagents were obtained through a material transfer agreement between the University of Torino and the indicated institution: WT-CFTR-CFBE41o- and F508del-CFTR-CFBE41o- (University of Alabama at Birmingham) and CFTR antibodies (University of North Carolina, Chapel Hill).

## Author contributions

AG and AM conceived and designed the overall study. AM and MM carried out the core of the experiments and analyzed the data. VC performed short-circuit current measurements in Ussing chambers. AL and AR performed the experiments using electron microscopy. PP performed the PLAs. ML performed the Endo H assay. KR performed the experiments using the syntaxin 5 plasmid. CT, DD, NP, and EH provided advice on the interpretation of data. AG and AM wrote the manuscript with input from co-authors. All authors reviewed and approved the final manuscript.

## Funding support

- Italian Cystic Fibrosis Research Foundation (FFC03/2022 to EH).
- Telethon Foundation (GGP20079 to AG).
- Italian Ministry of University and Research (PRIN 2022, grant 2022WHSCL3 to EH).
- AM was supported by a postdoctoral fellowship from the European Cystic Fibrosis Society and Cystic Fibrosis Europe.

## Supplementary Material

Supplemental data

Supplemental data set 1

Unedited blot and gel images

Supporting data values

## Version 1 03/23/2026

Electronic publication
