# Supplementary material for: Targeting PI3Kγ anchoring enhances CFTR membrane localization and modulator efficacy via PKD1
Source: JCI Insight. 2026 Mar 23;11(6):e198846. doi: 10.1172/jci.insight.198846 (PMC13043088; doi:10.1172/jci.insight.198846)
Supplement: Supplemental data [file jciinsight-11-198846-s230.pdf]

Supplementary Information

Targeting PI3Kγ Anchoring Enhances CFTR Membrane Localization and Modulator Efficacy via PKD1

Alessandra Murabito[1†], Marco Mergiotti[1†], Valeria Capurro[2], Alessia Loffreda[3], Mingchuan Li[1], Paola Peretto[1], Kai Ren[1], Andrea Raimondi[3], Carlo Tacchetti[3,4], Dario Diviani[5], Nicoletta Pedemonte[2], Emilio Hirsch[1,6] and Alessandra Ghigo[1,6*]

[1]Department of Molecular Biotechnology and Health Sciences, Molecular Biotechnology Center, University of Torino; Torino, Italy

[2]UOC Genetica Medica, IRCCS Istituto Giannina Gaslini; Genova, Italy

[3]Experimental Imaging Center, IRCCS Ospedale San Raffaele; Milan, Italy

[4]Università Vita-Salute San Raffaele, Milan, Italy

[5]Department of Biomedical Sciences, Faculty of Biology and Medicine, University of Lausanne; Lausanne, Switzerland

[6]Kither Biotech S.r.l.; Torino, Italy

†Equal contribution

*Corresponding authors: Alessandra Ghigo, Via Nizza 52, 10126, Torino, ITALY.

alessandra.ghigo@unito.it

Supplementary Figures

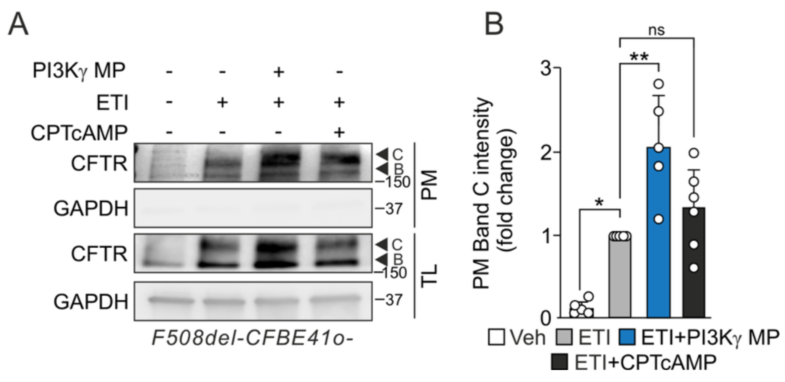

Fig S1. Global cAMP elevation via chronic CPTcAMP treatment does not reproduce the effect of PI3Kγ MP on CFTR trafficking

A) Representative Western blot of CFTR and GAPDH in PM fractions and total lysates (TL) of F508del-CFBE41o- cells treated for 24 h with ETI (3 µM VX-445, 10 µM VX-661, and 1 µM VX-770) alone or in combination with PI3Kγ MP (25 µM) or CPTcAMP (100 µM). GAPDH was absent from PM fractions and served as a loading control in TL.

B) Quantification of PM CFTR band C intensity as in (A), presented as fold change relative to ETI alone. *$P<0.05$ and **$P<0.01$ by one-way ANOVA with Bonferroni's post-hoc test. Data are mean ± SEM. Data points represent independent biological replicates.

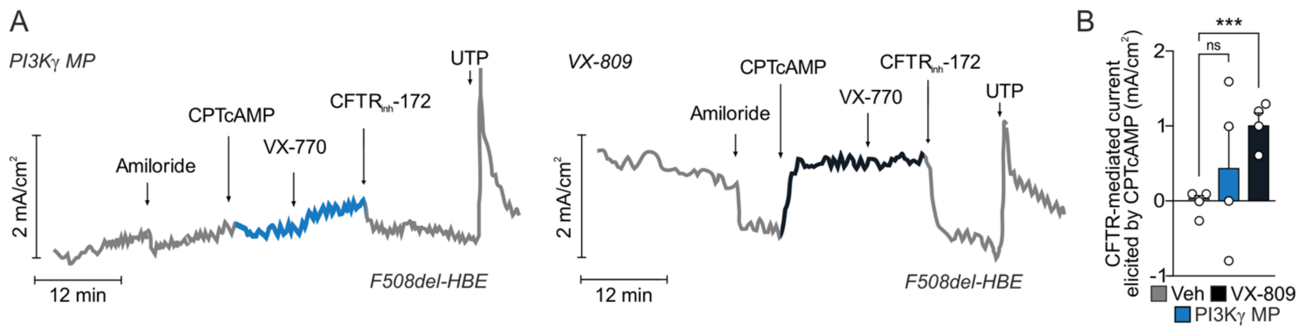

Fig S2. Chronic PI3Kγ MP treatment does not replicate the effect of the CFTR corrector VX-809

A) Representative short-circuit current (I_{SC}) traces in primary human bronchial epithelial (HBE) cells from a F508del/F508del CF donor grown at the air-liquid interface (ALI). Cells were treated for 24 hours with either PI3Kγ MP (10 μM), or the CFTR corrector VX-809 (3 μM), followed by sequential exposure to amiloride (10 μM), CPTcAMP (100 μM), VX-770 (1 μM), $CFTR_{inh}$-172 (10 μM), and UTP (100 μM).

B) Average CPTcAMP-stimulated current from n=4 technical replicates as in (A). ***P<0.001 by Student's t-test. Data are mean ± SEM.

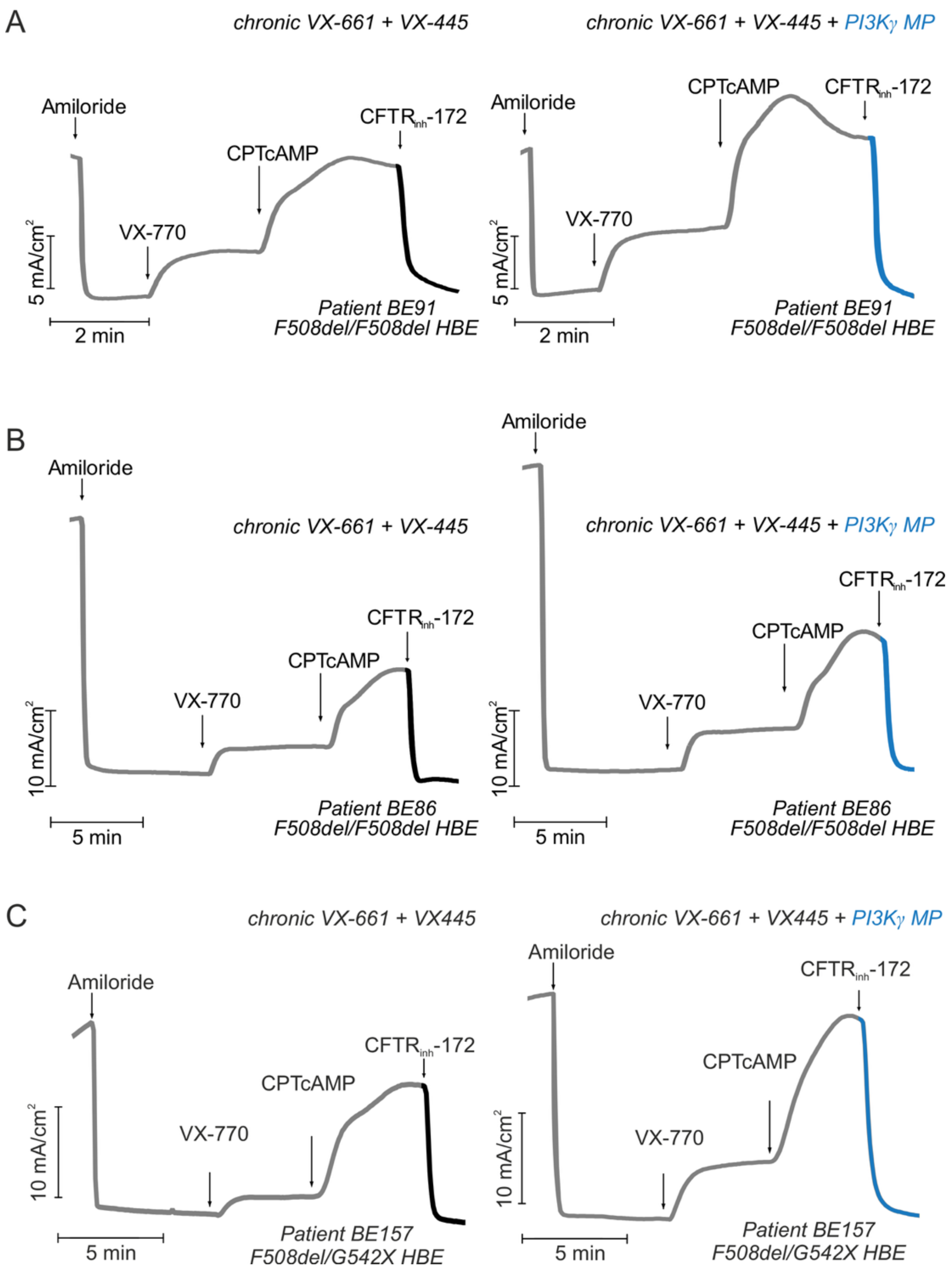

Fig. S3. Effects of PI3Kγ MP in human bronchial epithelial cells from different F508del/F508del and F508del/G542X CF donors

A-B) Representative short-circuit current (I_{SC}) traces in primary human bronchial epithelial (HBE) cells from two F508del/F508del CF donors (patient BE91 and BE86) grown at the air-liquid interface (ALI). Cells were corrected for 24 h with 10 μM VX-661 and 3 μM VX-445, with or without PI3Kγ MP (10 μM), followed by acute exposure to amiloride (100 μM), VX-770 (1 μM), CPTcAMP (100 μM), and $CFTR_{inh}$-172 (10 μM).

C) Representative I_{SC} traces in primary HBE cells from a F508del/G542X CF donor (patient BE157) grown at the ALI. Cells were corrected for 24 h with 10 μM VX-661 and 3 μM VX-445, with or without PI3Kγ MP (10 μM), followed by acute exposure to amiloride (100 μM), VX-770 (1 μM), CPTcAMP (100 μM), and $CFTR_{inh}$-172 (10 μM).
