# Supplementary material for: Targeting PI3Kγ anchoring enhances CFTR membrane localization and modulator efficacy via PKD1
Source: JCI Insight. 2026 Mar 23;11(6):e198846. doi: 10.1172/jci.insight.198846 (PMC13043088; doi:10.1172/jci.insight.198846)
Supplement: Unedited blot and gel images [file jciinsight-11-198846-s232.pdf]

Fig 1c

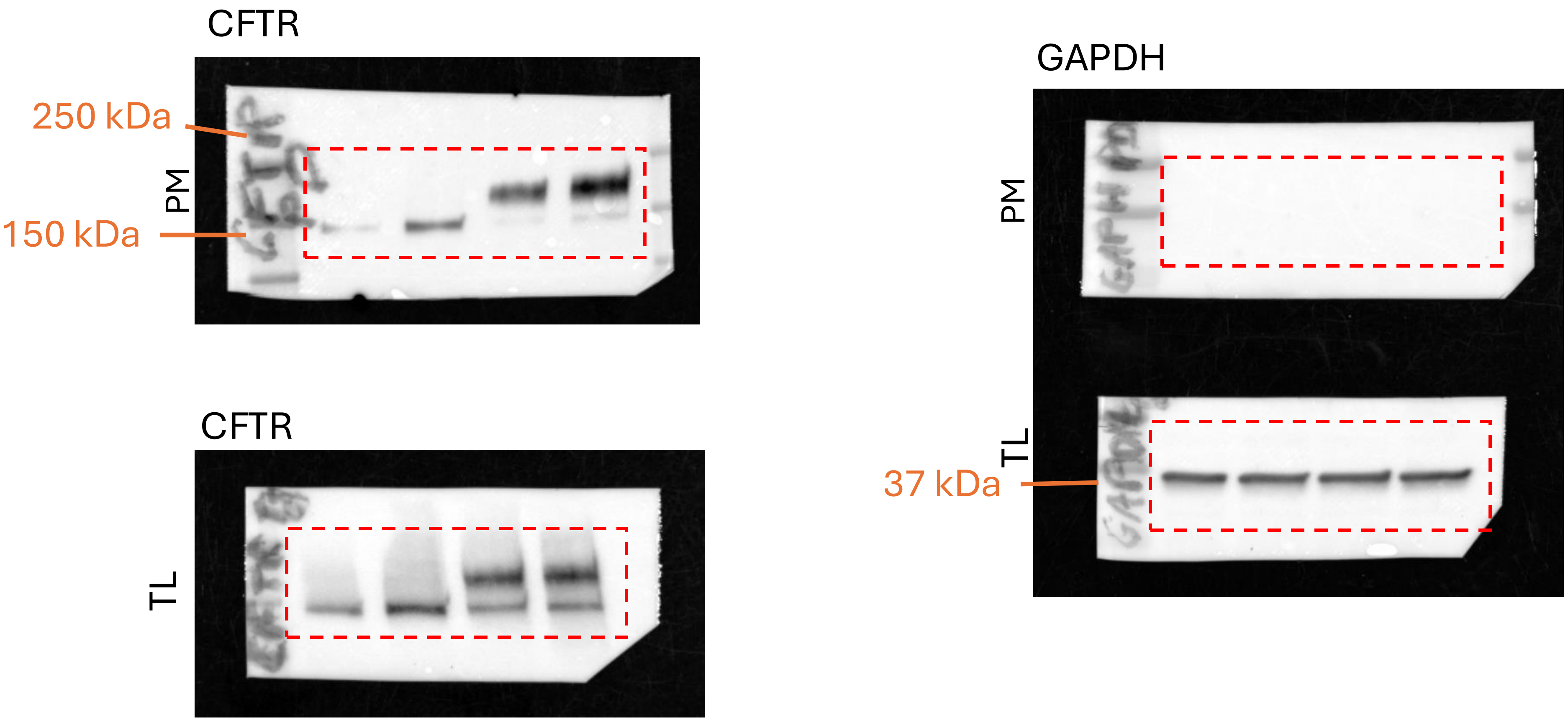

PM= Plasma Membrane
TL= Total lysates

Fig 1e

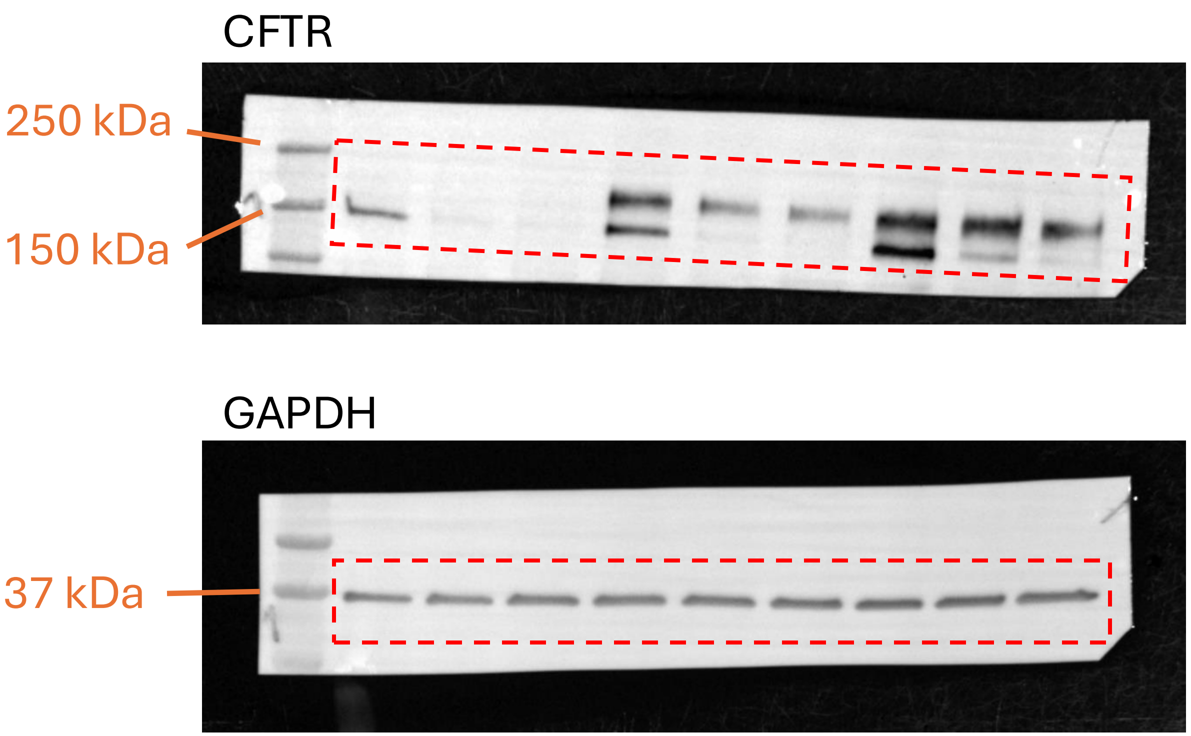

Fig 2c

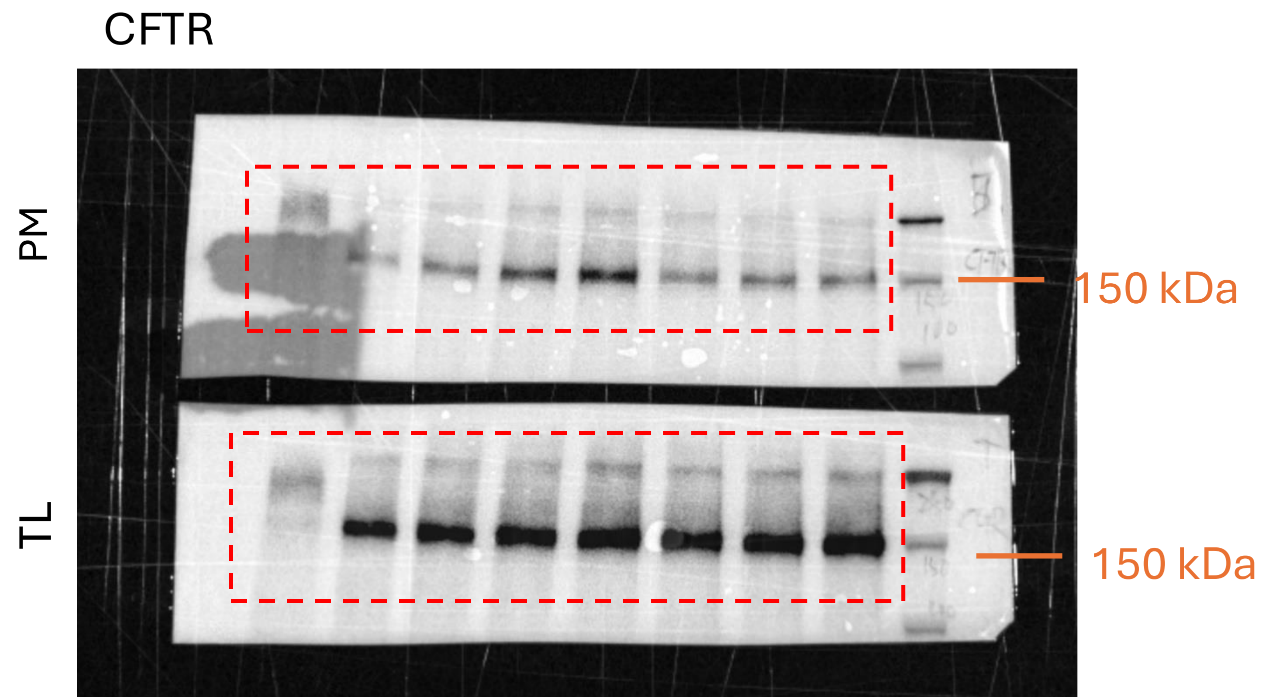

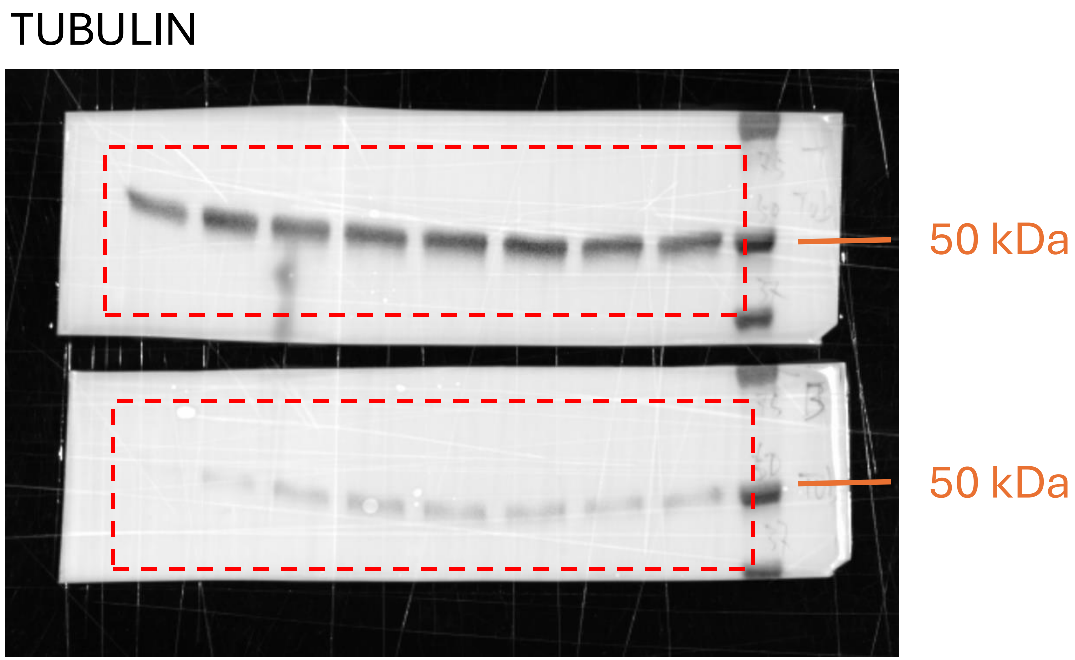

PM= Plasma Membrane
TL= Total lysates

Fig 2e

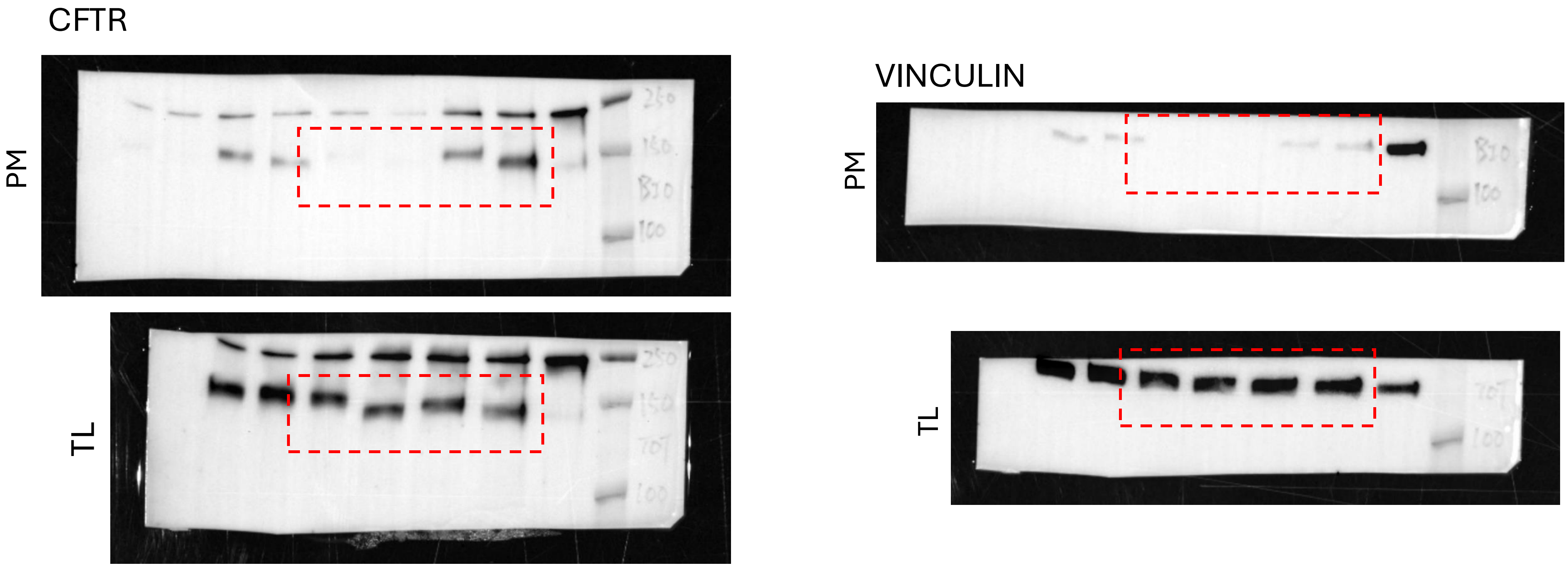

PM= Plasma Membrane
TL= Total lysates

Fig 4a

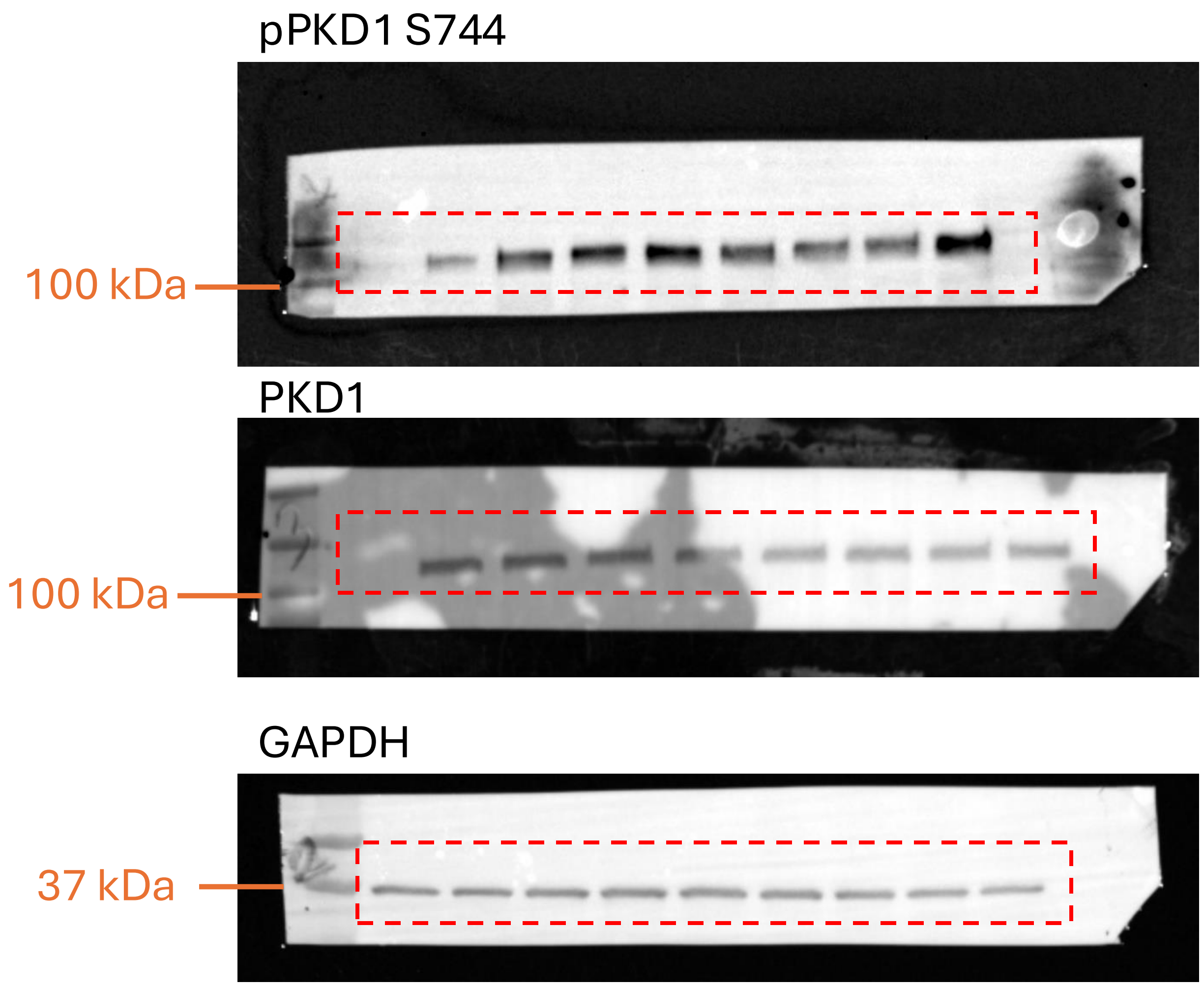

Fig 4d

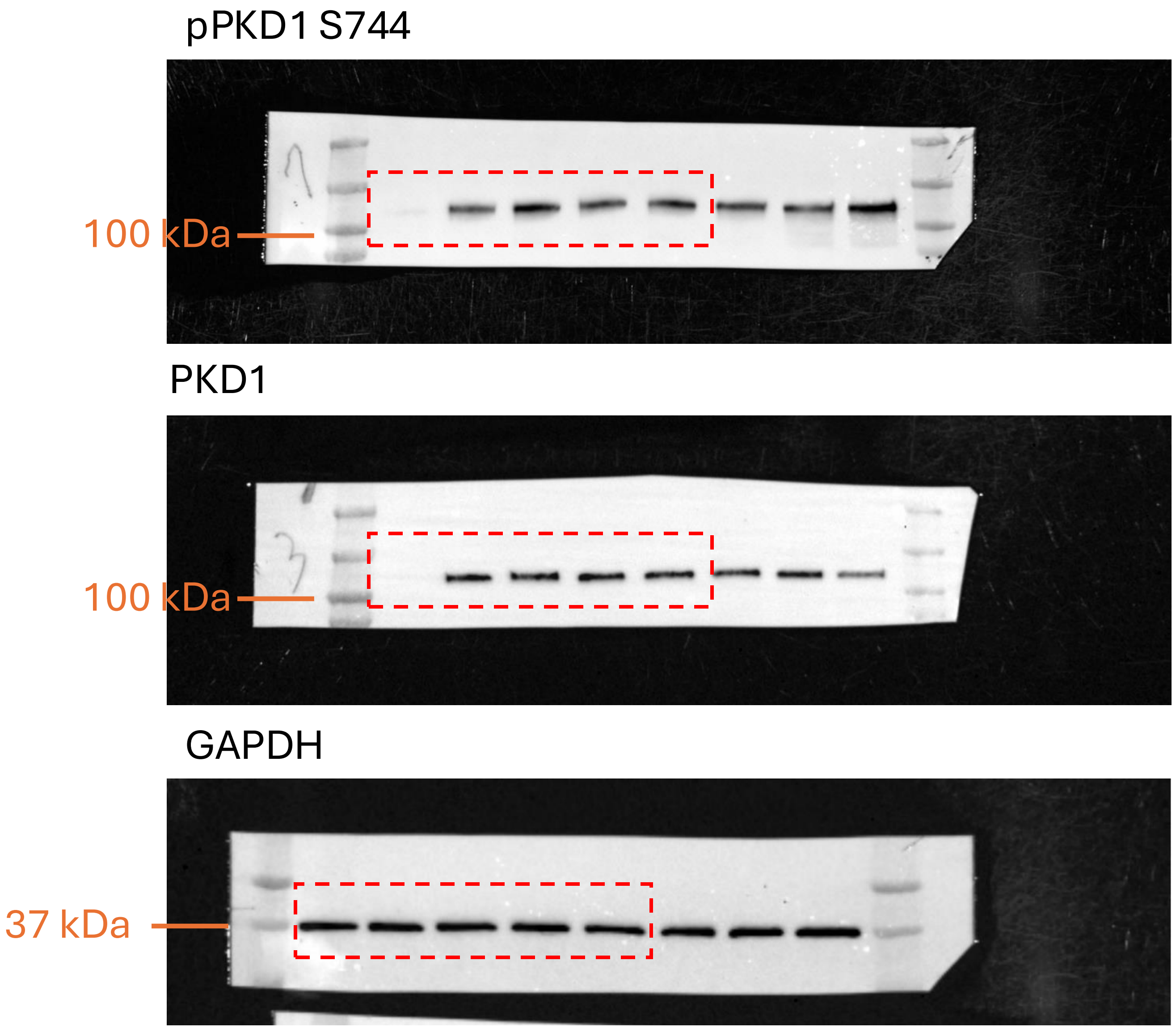

Fig 4f

Phospho-GFP-AKAP-Lbc

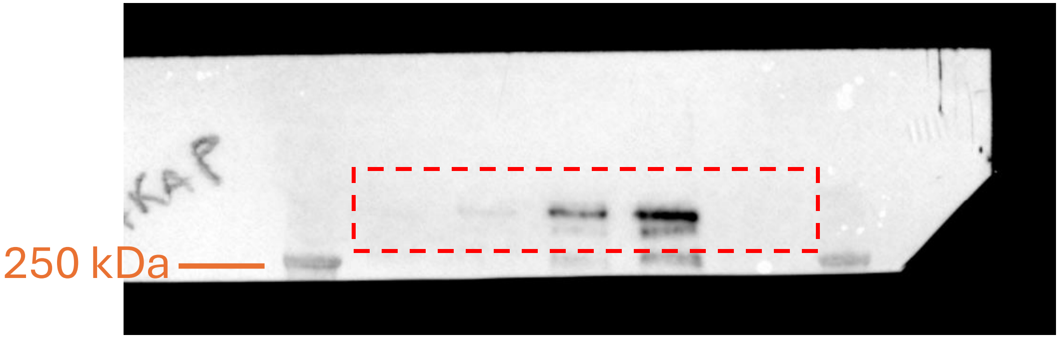

GFP-AKAP-Lbc

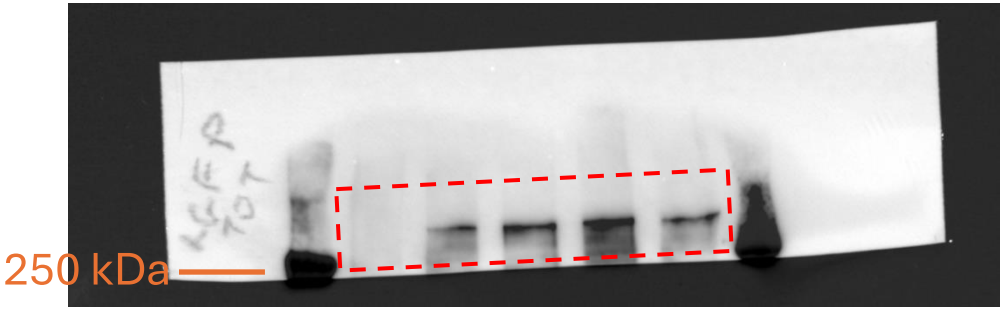

GAPDH

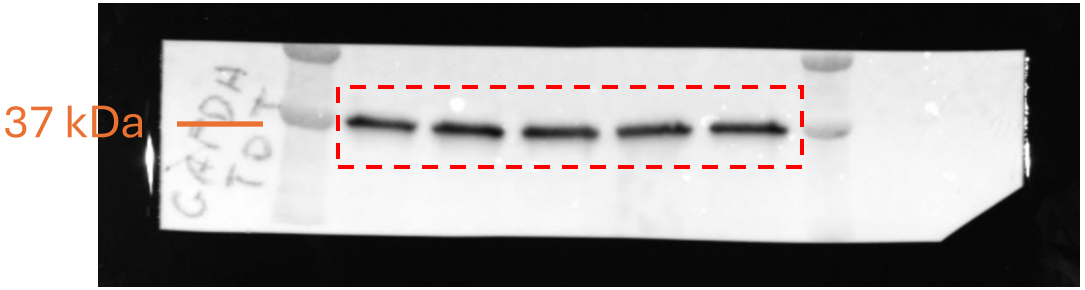

Fig 4h

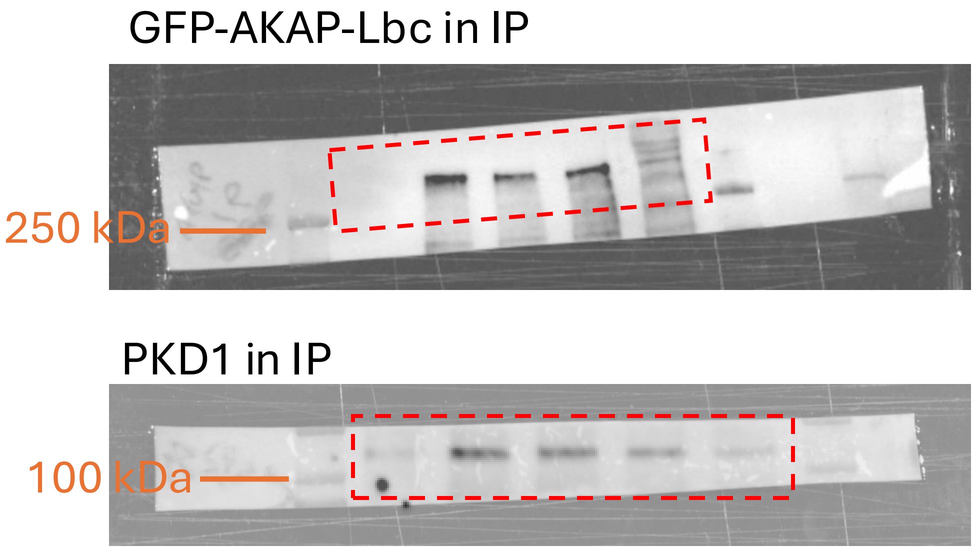
GFP-AKAP-Lbc in IP
250 kDa
PKD1 in IP
100 kDa

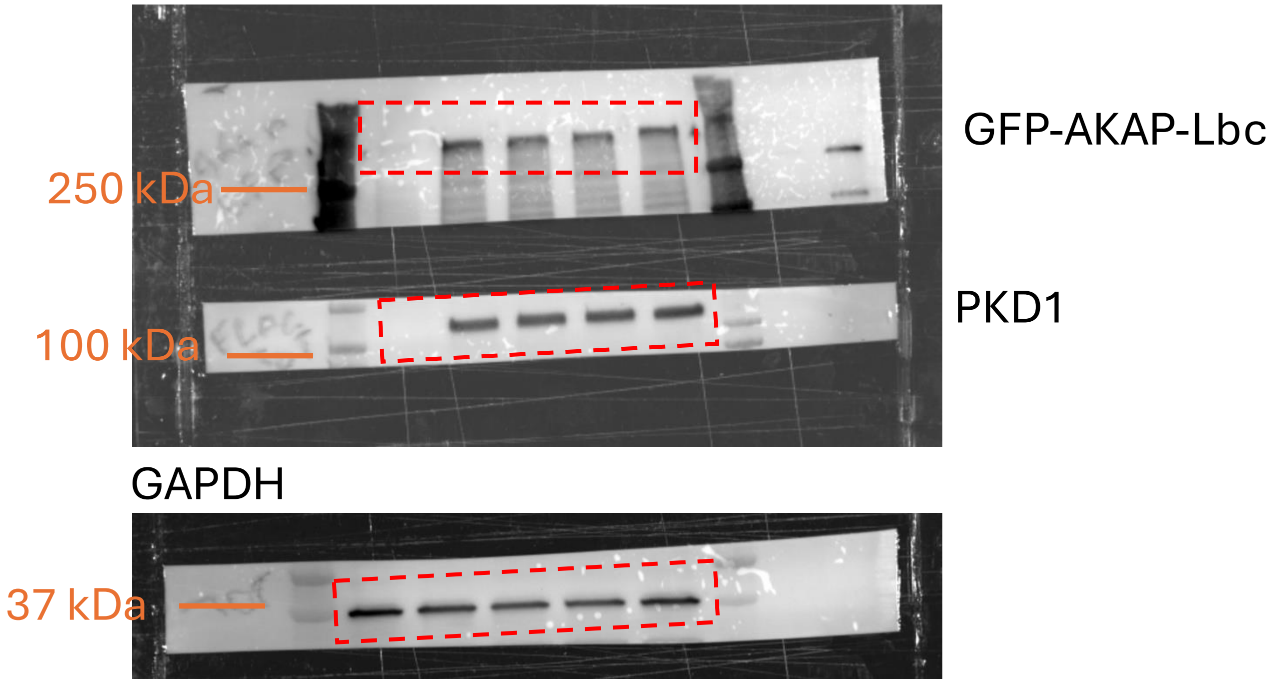
GFP-AKAP-Lbc
250 kDa
PKD1
100 kDa
GAPDH
37 kDa

Fig 4j

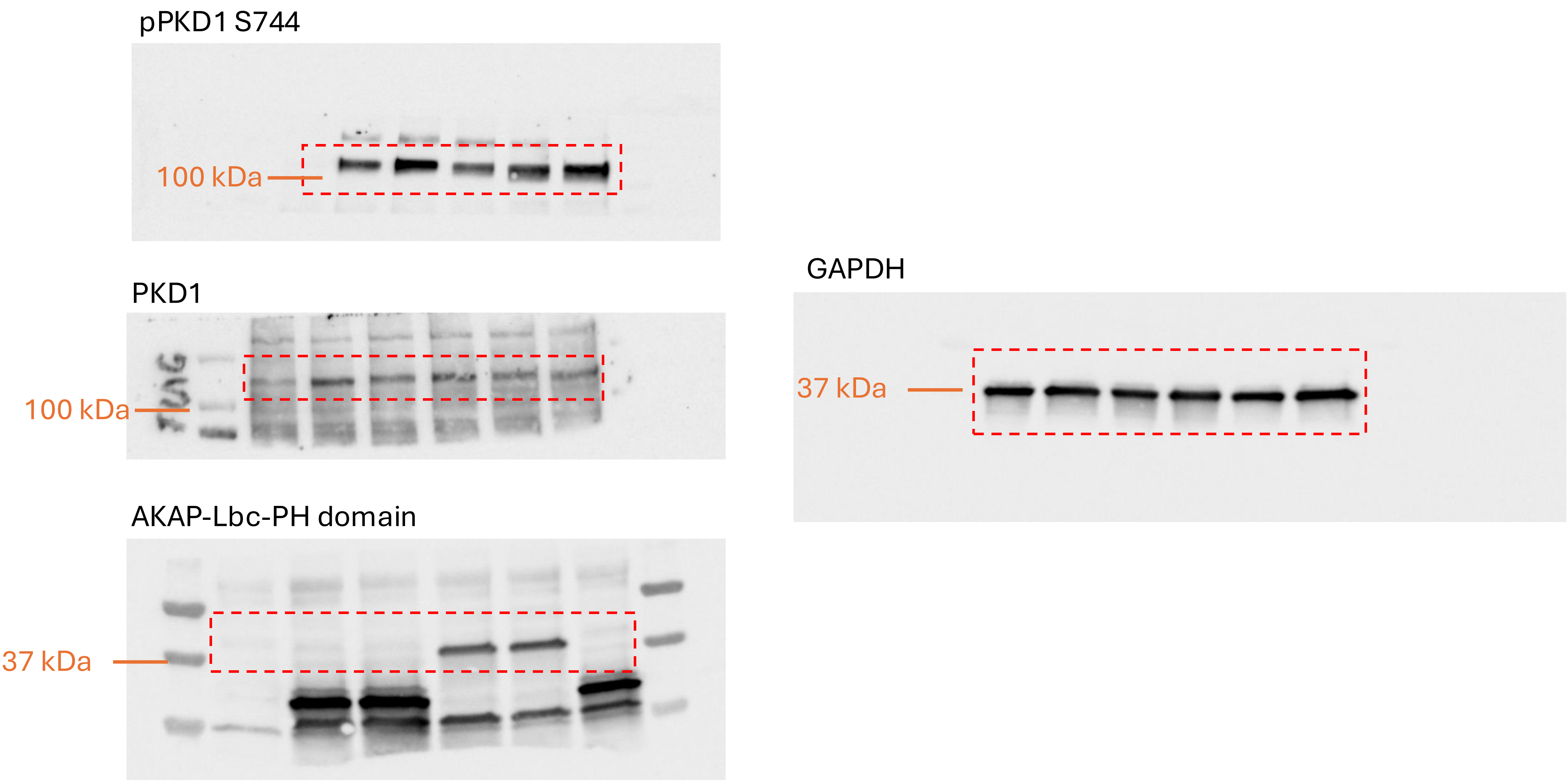
pPKD1 S744
100 kDa
PKD1
100 kDa
AKAP-Lbc-PH domain
37 kDa
GAPDH
37 kDa

Fig 5c

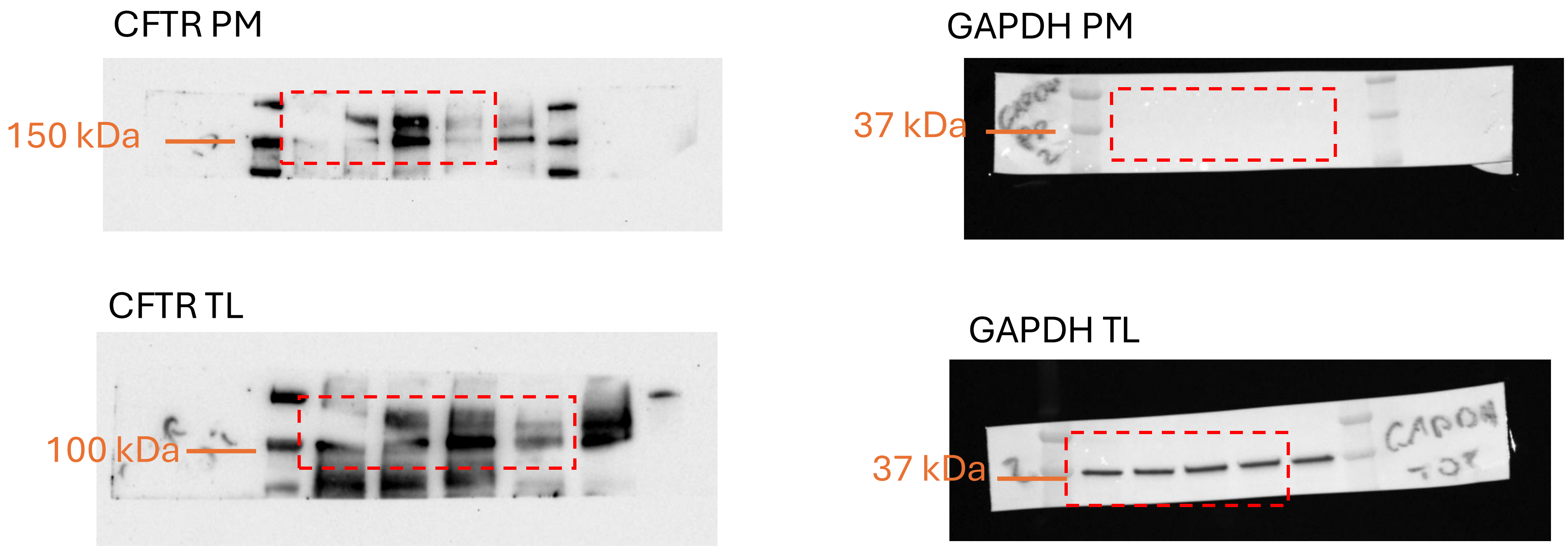

PM= Plasma Membrane
TL= Total lysates

Supplementary 1a

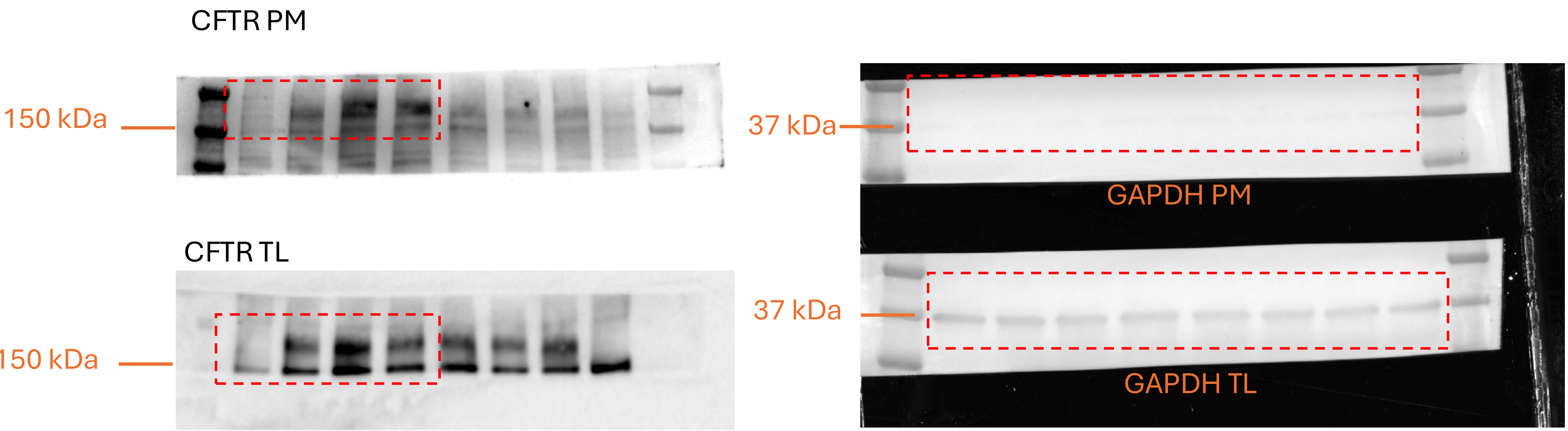

PM= Plasma Membrane
TL= Total lysates
